# Enhanced Electrochemical Detection of Lead Ions Using Schiff Base/MoS_2_ Modified Screen-Printed Electrodes

**DOI:** 10.1021/acsomega.5c07147

**Published:** 2025-11-11

**Authors:** Daniela Iannazzo, Zahra Akbari, Consuelo Celesti, Federica Bucolo, Salvatore V. Giofrè, Sebastiano Vasi, Dario Morganti, Khouloud Abid, Giovanni Neri

**Affiliations:** † Department of Engineering, 18980 University of Messina, 98166 Messina, Italy; ‡ Department CHIBIOFARAM, University of Messina, 98166 Messina, Italy; § Department MIFT, 18980 University of Messina, 98166 Messina, Italy; ∥ IMM CNR, 98166 Messina, Italy; ⊥ CNR ITAE, viale F. Stagno D’Alcontres 37, I-98156 Messina Italy

## Abstract

Environmental contamination by heavy metals (HMs) remains a critical concern; among them, Pb^2+^ is distinguished by its toxicity, bioaccumulation, and persistence. Here, we present disposable, noble-metal-free screen-printed carbon electrodes (SPCEs) functionalized with MoS_2_ nanosheets covalently conjugated to newly synthesized Schiff bases (SB1, SB2) via a monochloroacetic-acid linker, affording a chemically defined MoS_2_–SB interface for enhanced Pb^2+^ sensing in water. Using square-wave anodic stripping voltammetry (SWASV) under optimized conditions in PBS (pH 4.0), the MoS_2_@SB2/SPCE exhibits markedly higher currents than both bare and other modified electrodes. The sensor achieves an area-normalized sensitivity of 220.344 μA μM^–1^ cm^–2^, a limit of detection of 0.267 μM, and a primary linear range of 1–5 μM for Pb^2+^. This covalent interfacial design couples the high surface area and conductivity of MoS_2_ with SB2 driven chelation, yielding good selectivity, device-to-device reproducibility, and reliable performance in tap water samples. Our results outline a viable path for the low-cost and on-site monitoring of lead ions in complex water matrices.

## Graphical abstract

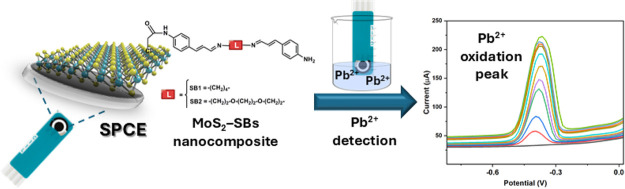

## 1 Introduction

Concerns related to environmental pollution from heavy metals [HMs] are well-known and continue to grow on a global scale.^1^ These pollutants, including lead (Pb), mercury (Hg), cadmium (Cd), nickel (Ni), copper (Cu), zinc (Zn), chromium (Cr), and arsenic (As), are recognized as particularly detrimental to the entire ecosystem and biologically unnecessary.^2^ Toxic HMs can enter the environment through both natural processes and human activities, ultimately accumulating in soils, water bodies, or the atmosphere. Mining, electroplating, smelting, fertilizer production, pesticide application, tanneries, paper, and electronics industries have contributed to significant releases of HMs into the natural ecosystem, which have been shown to disrupt physiological functions in biological systems.^3^ Furthermore, environmental contamination from heavy metals also arises from processes such as leaching, corrosion, atmospheric deposition, sediment resuspension into soil and groundwater, and metal evaporation from water sources.^4,5^ The toxicity of these hazardous materials depends on the specific type of heavy metals, as well as the concentration and duration of exposure to living organisms. The detection of HMs ions in environmental and biological systems is crucial because of their toxicity, potential for bioaccumulation, and persistence in the environment.^6^ Among these, lead is a nonessential heavy metal with unknown functions in the human body, classified by the International Agency for Research on Cancer (IARC) as carcinogenic, or potentially carcinogenic to humans.^7^ Exposure to Pb^2+^ ion is related to cancer and infections in various organs, including the urinary tract, reproductive system, central nervous system, and respiratory system.^8,9^ The maximum limit of Pb^2+^ ion in the human body is 0.015 mg/L.^10^ Consequently, due to its high toxicity, potential for bioaccumulation, and persistence in the environment, rigorous monitoring of this ion in both environmental and biological systems is essential. Traditional detection methods, such as atomic absorption spectroscopy (AAS) and inductively coupled plasma mass spectrometry (ICP–MS), provide high sensitivity and precision but are costly, require extensive sample preparation, and are not suitable for rapid, on-site monitoring.^11^ To overcome these limitations, electrochemical sensors based on screen-printed electrodes (SPEs) have emerged as a promising alternative for real-time and cost-effective heavy metal detection. SPEs offer several advantages, including ease of fabrication, portability, reproducibility, and compatibility with various electrode modifications.^12,13^ The chemical modification of nanomaterials and/or sensing elements onto SPEs further enhances their electrochemical performance, increasing sensitivity and selectivity toward target analytes.^14–16^

A wide variety of modified electrodes have been proposed for the electrochemical detection of HMs, highlighting the role of surface chemistry and nanostructure design in determining analytical performance. For instance, zinc oxide nanostructured films combined with Nafion have been explored for the detection of Pb^2+^ and Cu^2+^ ions, demonstrating good reproducibility.^17^ Similarly, Bi–Fe–carbon xerogel composites have shown extremely low detection limits for Pb^2+^ thanks to the synergistic redox activity of bismuth and the conductive carbon network.^18^ Moreover, green-synthesized ZnO nanoparticles integrated into carbon paste electrodes have been explored for Cu^2+^ detection.^19^ These studies collectively underline the importance of tailoring nanomaterial morphology, composition, and interface properties to achieve enhanced sensitivity and selectivity, motivating the rational design of hybrid systems such as the MoS_2_–Schiff base platform here reported.

In this work, we selected carbon SPCEs as the transducer because they are directly compatible with our MoS_2_–COOH/amide-coupling chemistry, provide low and stable backgrounds under SWASV that favor narrow, well-resolved stripping peaks, and offer robust batch-to-batch reproducibility at low cost for single-use measurements; given that the sensitivity gain is delivered by the MoS_2_–SB overlayer, SPCEs offer a pragmatic and effective choice over carbon-nanofiber or graphene SPEs. In recent years, two-dimensional (2D) inorganic nanomaterials, including transition metal dichalcogenides (TMDCs), have garnered significant attention in the electrochemical field due to their unique and remarkable properties.^20^ As semiconductors of the type MX_2_, where M refers to a transition metal atom (like molybdenum (Mo) or tungsten (W)) and X represents a chalcogen atom (such as sulfur (S), selenide (Se), or telluride (Te)). These nanosheets (NS) display a unique blend of characteristics, including a direct band gap, strong spin–orbit coupling, and advantageous electronic and mechanical properties, especially attractive for applications in advanced electronics, spintronics, optoelectronics, energy harvesting, flexible electronics, and sensors, among others.^21^ Molybdenum disulfide (MoS_2_) is a two-dimensional (2D) TMDC with a layered structure, where molybdenum (Mo) atoms are sandwiched between two layers of sulfur (S).^22^ Featuring a tunable bandgap (∼1.8 eV for monolayers), high surface area, and remarkable structural, electronic, and catalytic properties.^23,24^ Moreover, MoS_2_ is an ideal material for electrochemical sensing.^25^ Indeed, MoS_2_-based electrochemical sensors have been investigated for the detection of a wide range of biomolecules,^26,27^ and heavy metals.^28^

MoS_2_-nanosheets (MoS_2_–NS) functionalized with monochloroacetic acid (MCA) have been employed as immunosensors, by exploiting the ability of chlorine atoms to occupy sulfur vacancies in MoS_2_. The resulting MoS_2_–COOH composite was covalently linked to the amino groups of the peptides, enhancing its sensitivity and affinity, and making it highly effective for immune-enzymatic detection.^29^ In this study we exploited the improved selectivity of MoS_2_–NS toward Pb^2+^ ions by synthesizing and conjugating MoS_2_–COOH with newly synthesized Schiff Bases (SBs). SBs are known for their strong coordination ability with metal ions due to the presence of electron-donating nitrogen (−C=N) and oxygen groups, which facilitate selective binding with HMs.^30^ Their integration with two-dimensional (2D) materials such as MoS_2_ represents a promising strategy to enhance sensing performance. The SBs used in this study were chosen based on previous Molecular Dynamics (MD) and Density Functional Theory (DFT) analyses of newly designed SBs, where we assessed their complexation abilities with toxic metal ions. Each of the designed SBs contained a free amino group, essential for forming an amide bond with the MoS_2_–COOH composite.^31^

This work reports, for the first time, a MoS_2_–Schiff base (SB1/SB2) interface constructed via amide coupling to MoS_2_–COOH and rationally designed (guided by MD/DFT). By replacing weak physisorption with a robust, chemically defined molecular layer, the platform couples targeted recognition with efficient charge transfer, achieving higher Pb^2+^ preconcentration, well-resolved Pb^2+^/Cd^2+^ peaks, high device-to-device reproducibility, and reliable operation in tap water. The architecture is free of noble metals and implemented through a short, low-cost, scalable workflow that is inherently compatible with disposable devices.

Given the promising theoretical affinity of both SBs and in particular of SB2 for Pb^2+^ ion, the so obtained MoS_2_–SB1 and MoS_2_–SB2 nanocomposites (Figure 1) were fully characterized and loaded on the carbon working electrodes of SPCEs to evaluate their ability to sensitively and selectively detect Pb^2+^ ion using square wave anodic stripping voltammetry (SWASV).

**Figure 1:**
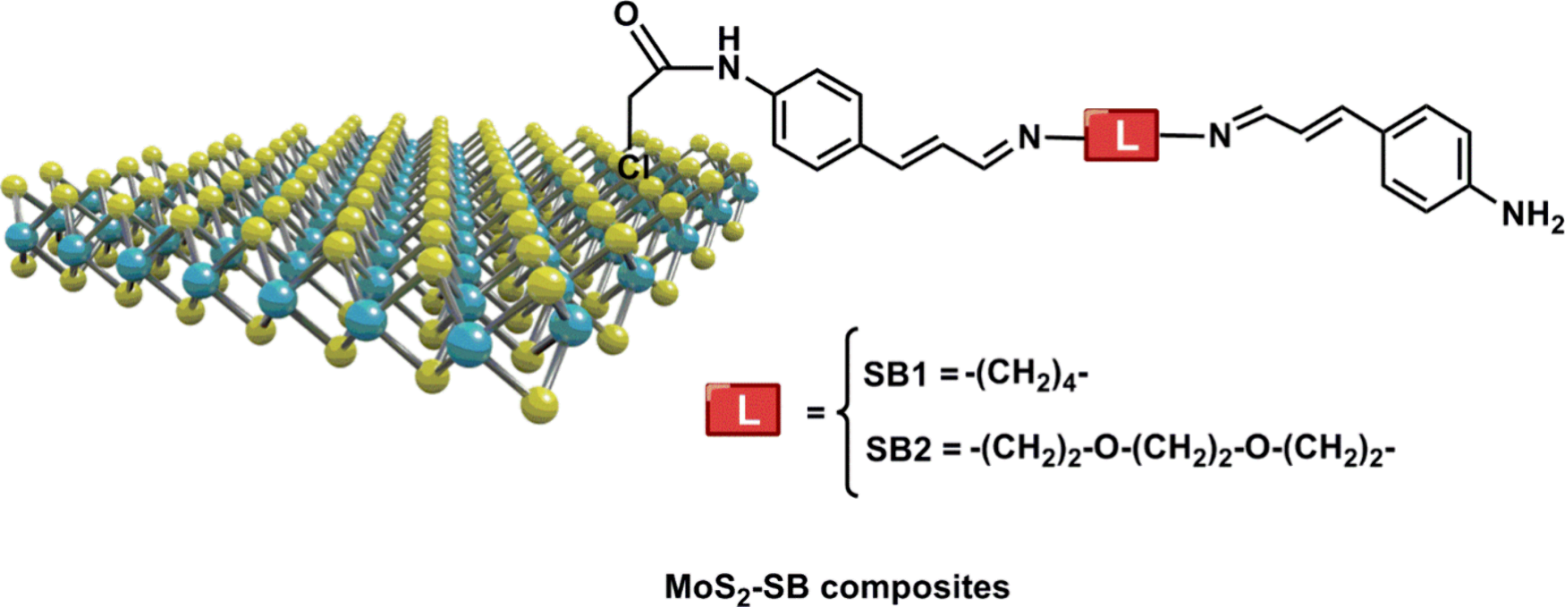
MoS_2_–SBs for HMs electrochemical detection.

## 2 Experimental Section

### 2.1 Materials

All reagents and solvents employed in the synthesis of SBs and of composite materials were obtained from Sigma-Aldrich (St. Louis, MO, USA), of analytical grade, and were used directly without additional purification. MoS_2_–NS were obtained from bulk MoS_2_ powder with a purity 99%. Lead nitrate with purity ≥ 99.95% was employed to prepare aqueous solutions of ion. The acetate buffer was prepared by adjusting the pH of a 0.1 M acetic acid solution with NaOH. Double-distilled water was used throughout the experimental procedures. Screen printed carbon electrodes (SPCE) were purchased from Metrohm-DropSens (www.dropsens.com).

### 2.2 Chemical Characterization

Nuclear magnetic resonance (NMR) spectra were recorded at 500 MHz using a Varian spectrometer (Agilent Technologies, Palo Alto, CA, USA); chemical shifts are given in parts per million, using trimethylsilane (TMS) as the internal standard. Thin-layer chromatography was conducted on aluminum plates coated with Merck 60-F254 silica gel. Preparative separations were performed via flash column chromatography using Merck silica gel with a particle size range of 0.063–0.200 mm. Infrared spectra were recorded using a Fourier-Transform Infrared (FT-IR) Spectrum Two FT-IR Spectrometer (PerkinElmer Inc., Waltham, MA, USA) by the ATR method in the range of 4000–500 cm^–1^. Ultraviolet–visible (UV–vis) spectra were obtained in the 200–450 nm spectral range by using an employing 1 cm rectangular quartz cells. Thermogravimetric analysis (TGA) were performed using a PerkinElmer TGA 4000 instrument (PerkinElmer Inc., Waltham, MA, USA). These measurements were conducted in an inert atmosphere using an argon flow of 20 mL/min with a scan rate of 10 °C/min and in the temperature range of 25–1000 °C. Samples were weighed in alumina crucibles, using sample amounts in the range of 1–2 mg. UV–vis spectroscopy was acquired using a Lambda 365 UV–Vis spectrophotometer from PerkinElmer within the wavelength range of 200–800 nm, under ambient air conditions at room temperature (PerkinElmer Inc., Waltham, MA, USA). Zeta potential measurements were accomplished using the Zeta sizer 3000 instrument (Malvern Instruments Ltd., Worcestershire, United Kingdom.). The Raman measurements were performed by drop-casting sample solutions onto a Raman-grade CaF_2_ commercial slide as a support substrate to avoid any spurious fluorescence contribution, as it is characterized by a very low amount of Raman peaks. The backscattered Raman signals were collected by using a LabRam HR-EVO Horiba operating at 532 nm, 50X LWD (long working distance) with a laser power of 0.8 mW per μm^2^ and equipped with a CCD Syncerity Horiba and NRS 7200 microRaman spectrometer with 532 nm wavelength and applied power of 6.5 mW. In particular, the micro-Raman spectrometer was used for the evaluation of the presence of the exfoliated MoS_2_ in the original sample and the samples of MoS_2_ treated with MCA and combined with Schiff bases. Scanning electron microscopy images (SEM) were performed with an FEI Quanta 450 FEG instrument (Thermo Fisher Scientific, Hillsboro, OR, USA) at room temperature and operating in high vacuum, at 20 kV, using an Everhart–Thornley detector (ETD). Energy-dispersive X-ray (EDX) analyses were carried out using the detector Octane Plus Silicon Drift (Ametek, Berwyn, PA, USA), equipped with a 30 mm2 super ultrathin window (SUTW). HM concentrations were determined using an Agilent 7500cx ICP–MS (Agilent Technologies, Santa Clara, CA, USA). To suppress polyatomic interferences originating from the plasma and sample matrix, the instrument operated with an octopole collision/reaction cell using helium. Operating parameters were: RF power 1500 W; plasma gas flow 15 L min^–1^; auxiliary gas 0.9 L min^–1^; carrier gas 1.1 L min^–1^; He collision gas 4 mL min^–1^; spray-chamber temperature 2 °C; sampling depth 9 mm; sample uptake 1 mL min^–1^; nebulizer pump 0.1 rps; extraction-lens voltage 1.5 V.

### 2.3 Synthesis of Schiff Bases

#### 2.3.1 Reduction of *trans*-4-Nitrocinnamaldehyde with Fe­(OH)_3_

A hot solution of 2 g of 4-nitrocinnamaldehyde in 150 mL ethanol was dropped into a hot mixture of 20 g FeSO_4_·7H_2_O, 150 mL water and 150 mL concentrated aqueous ammonia, and the mixture was heated to reflux for 1 h; the reaction mixture was allowed to cool to about 40°, then extracted four times with 50 mL ether. The ether extract was concentrated under reduced pressure to obtain 4-aminocinnamaldehyde (Figure 1). ^1^H NMR (500 MHz, Chloroform-*d*) δ = 9.61 (d, *J* = 7.9 Hz, 1H), 7.39 (d, *J* = 8.5 Hz, 2H), 7.36 (d, *J* = 15.8 Hz, 1H), 6.68 (d, *J* = 8.5 Hz, 2H), 6.55 (dd, *J* = 15.8, 7.9 Hz, 1H), 4.07 (bs, 2H).

#### 2.3.2 Synthesis of SB1 and SB2

0.40 mmol of 4-aminocinnamaldehyde was reacted with 0.20 mmol of 1,4-Diaminobutane, or 0,20 mmol of 2,2′-(Ethylenedioxy)­bis­(ethylamine), with a catalytic amount of magnesium sulfate in CH_2_Cl_2_ for 12 h at room temperature. The resulting compound was evaporated under reduced pressure and purified by 98:2 CH_2_Cl_2_:CH_3_OH chromatographic column, giving the compound SB1 and SB2, respectively (Figure 2).

**Figure 2:**
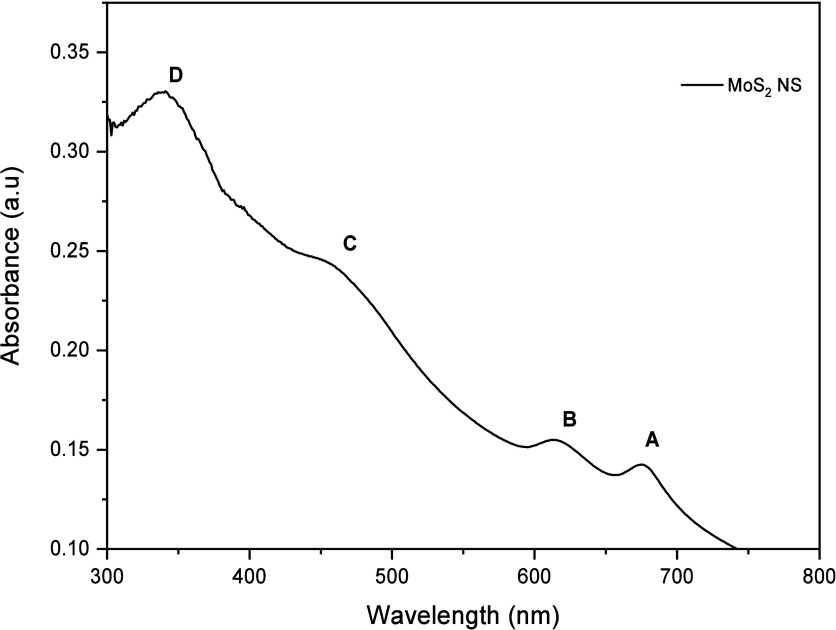
UV–Vis absorption spectrum of exfoliated MoS_2_–NS in deionized water at concentration of 0.1 mg/mL.

##### 2.3.2.1 4,4′-((1E,1′E,3Z,3′E)-(butane-1,4-diylbis­(azaneylylidene))­bis­(prop-1-en-1-yl-3-ylidene))­dianiline (SB1)

^1^H NMR (500 MHz, Chloroform-*d*) δ = 7.96 (d, *J* = 8.7 Hz, 2H), 7.27 (d, *J* = 8.2 Hz, 4H), 6.82 (dd, *J* = 15.9, 5,5 Hz, 2H), 6.71 (dd, *J* = 15.9, 8.7 Hz, 2H), 6.64 (d, *J* = 8.6 Hz, 4H), 3.84 (bs, 4H), 3.55–3.45 (m, 4H), 1.73–1.67 (m, 4H). ^13^C NMR (126 MHz, Chloroform-*d*) δ = 163.34, 147.63, 141.82, 128.87, 126.47, 124.66, 115.12, 61.33, 28.89.

##### 2.3.2.2 4,4′-((1E,3E,13E,15E)-7,10-dioxa-4,13-diazahexadeca-1,3,13,15-tetraene-1,16-diyl)­dianiline (SB2)

^1^H NMR (500 MHz, Chloroform-*d*) δ = 7.97 (d, *J* = 8.7 Hz, 2H), 7.27 (d, *J* = 8.6 Hz, 4H), 6.84 (d, *J* = 15.9 Hz, 1H), 6.82 (d, *J* = 15.9 Hz, 1H), 6.71 (dd, *J* = 15.9, 8.7 Hz, 2H), 3.87 (bs, 4H), 3.74–3.70 (m, 4H), 3.68–3.60 (m, 8H). ^13^C NMR (126 MHz, Chloroform-*d*) δ = 165.02, 147.78, 142.35, 128.94, 126.28, 124.44, 115.08, 73.53, 71.16, 70.57, 60.83.

#### 2.3.3 Synthesis of MoS_2_–COOH Nanocomposite

The first synthetic step involved exfoliating MoS_2_ powder. In this procedure, 30 mg of MoS_2_ powder was added to a 30 mL quartz vessel containing 30 mL of *N*-methylpyrrolidone (NMP) as solvent. The reaction was carried out under a nitrogen atmosphere with high-speed stirring to ensure uniform mixing, while the system was exposed to microwave irradiation at 140 W for 1 h. Following the reaction, the mixture was centrifuged at 1500 rpm for 20 min to remove larger aggregates. The resulting supernatant was characterized by UV–vis spectroscopy and used in subsequent reactions. For the second step, 7.5 mL of the MoS_2_ solution in NMP (at a concentration of 1 mg/mL) was combined with 500 mg of sodium hydroxide (NaOH) and 500 mg of monochloroacetic acid (MCA). The mixture was stirred under ultrasound for 3 h, followed by overnight stirring at room temperature. The mixture was then centrifuged several times, and the supernatant was replaced with deionized water. The final product, a carboxylated MoS_2_ suspension modified with monochloroacetic acid, was obtained and referred to as MoS_2_–COOH.

#### 2.3.4 Synthesis of MoS_2_–SB1 and MoS_2_–SB2

For the functionalization of MoS_2_–COOH, 0.04 mmol of hydroxybenzotriazole (HOBt) and 0.04 mmol of EDC (1-ethyl-3-(3-(dimethylamino)­propyl) carbodiimide) were used in a 1:1 molar ratio. The EDC/HOBt solution was added to the MoS_2_–COOH dispersion to activate the −COOH groups at room temperature for 15 min. Following this, 0.04 mmol of either SB1 or SB2 were added, and the reaction was allowed to stir for 48 h at room temperature. Afterward, the reaction mixture was diluted with deionized water and purified by dialysis using a membrane with a molecular weight cutoff (MWCO) of 12,000 Da for 8 h. The final products, MoS_2_–SB1 and MoS_2_–SB2, were obtained after purification.

### 2.4 Electrochemical Studies

Electrochemical tests were performed using commercial screen-printed carbon electrodes (SPCE) (Dropsens, Spain). The modification of the bare electrode was achieved via drop casting of 10 μL of MoS_2_-MCA-SB into the working electrode surface (WE, 4 mm diameter) at room temperature. Electrochemical characterization of the modified electrode included cyclic voltammetry (CV) and electrochemical impedance spectroscopy (EIS) in ferrocyanide solution ([Fe­(CN)_6_]^3–^/^4–^) across a frequency range of 10^5^–10^–2^ Hz using DropSens μStat 400 and Autolab PGSTAT204 systems. Data acquisition was performed using Dropview 8400 and Nova 2.1 software. Square wave anodic stripping voltammetry (SWASV) curves were recorded under optimized parameters: frequency = 10 Hz, step potential (Estep) = 0.016 V, square wave amplitude (Eamp) = 0.025 V, deposition potential (Edep) = −1 V, and deposition time (tdep) = 180 s, in PBS at pH = 4. The real sample analyses have ben performed using the standard addition method. Briefly, tap water aliquots were adjusted to pH 4.0 with PBS, transferred to the electrochemical cell, and analyzed by SWASV under the same electrochemical parameters optimized in buffer. After acquiring the unspiked signal, we performed 3–4 on-cell standard additions of Pb^2+^, running a full SWASV scan after each addition. Each sample was analyzed with *n* = 3 independently fabricated electrodes.

## 3 Results and Discussion

### 3.1 Synthesis of the Sensing Material

The SBs used in this study as sensing elements for the electrochemical detection of Pb^2+^ were chosen based on previous computational results.^31^ From this study, SB1 and SB2 (Scheme 1) emerged as promising ligands for HM complexation through the N-imino moieties or, in the case of SB2, assisted by the oxygen atoms in the ethylene diether chain. Notably, SB2 displayed the most favorable interaction with Pb^2+^ ions. The synthetic strategy toward the formation of SB1 and SB2, here reported for the first time, involved the reduction of trans-4 nitrocinnamaldehyde **1** to the corresponding amino derivative **2** essential for subsequent conjugation with the MoS_2_-based composite (Scheme 1). The reduction reaction was carried out using a ferrous salt (FeSO_4_) as the reducing agent in an ethanol–water mixture with aqueous ammonia. The subsequent extraction with diethyl ether gave the corresponding aromatic amine in almost quantitative yield, as also reported in the literature for the reduction of other aromatic p-nitro aldehydes.^32^ The reaction of this aldehyde, whose structure was confirmed by ^1^H NMR spectroscopy (Figure S1, ESI) with 1,4-diaminobutane or 2,2′-(ethylenedioxy)­bis­(ethylamine) in dichloromethane, at room temperature, quantitatively yielded the corresponding SB1 and SB2. The obtained SBs were characterized by ^1^H and ^13^C NMR (see Figures S2–S5, ESI). As confirmed by the disappearance of the aldehyde signal at 9.61 ppm in the ^1^H NMR spectrum (Figure S1, ESI), and by the presence of diagnostic imine and alkene proton signals in the expected regions (Figures S2–S5), the successful formation of SB1 and SB2 was validated. The ^1^H NMR spectra of both compounds show the disappearance of the proton at 9.61 ppm of the starting aldehyde, the presence of the imine (−CH=N−) protons as doublets at 7.96 and 7.97 for SB1 and SB2, respectively, and the resonances of the olefinic protons in the 6.7–7.0 ppm region. Additionally, the ^1^H NMR spectrum of SB1 shows the characteristic methylene (−CH_2_−) signals of the 1,4-butane bridge as multiplets centered at δ 3.84, 3.50, and 1.70 ppm (Figure S2, ESI). For SB2, the spectrum exhibits resonances for the ethylene dioxy (−OCH_2_–CH_2_O−) and ethylene (−CH_2_–CH_2_−) groups as multiplets centered at δ 3.87, 3.72, and 3.64 ppm (Figure S3, ESI). The ^13^C NMR spectra of both samples show the presence of the diagnostic peaks of imine (−CH=N−) carbons at 163.34 and 165.02 ppm for SB1 and SB2, respectively, and the resonances of aromatic structures and alkenyl moieties in the range of 110–140 ppm (Figures S4 and S5, ESI).

**Scheme 1:**
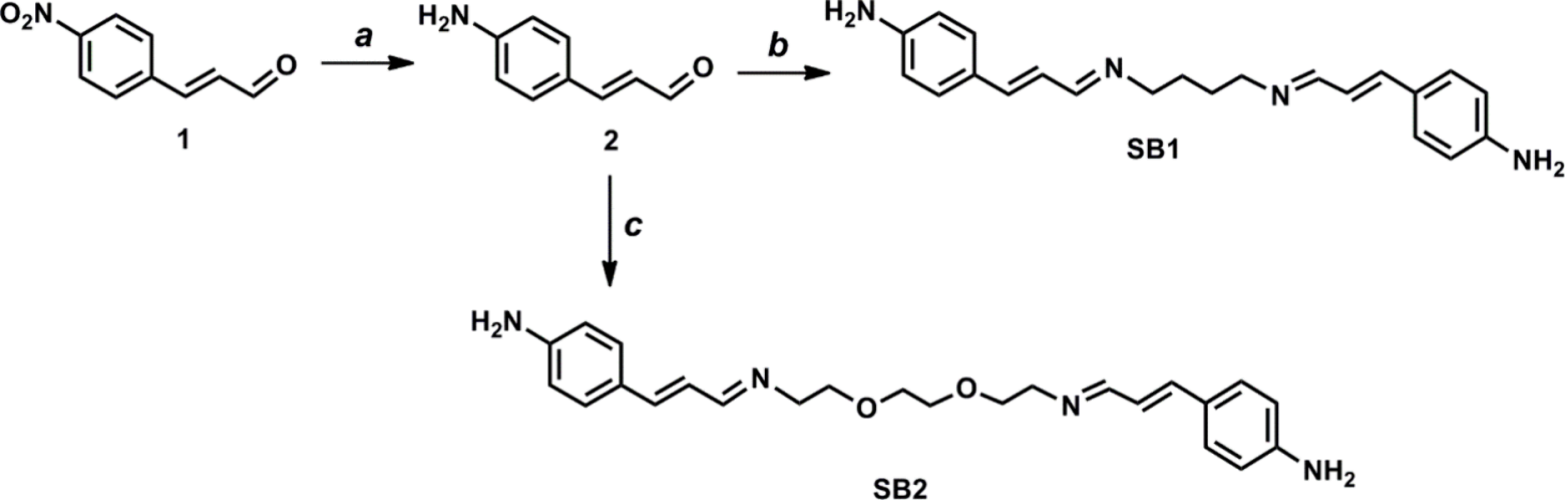
*Reagents and Condition:* (a) Ethanol, FeSO_4_·7H_2_O, H_2_O, NH_4_OH Conc., Reflux 1 h, Yield 98%; (b) 1,4-Diaminobutane, CH_2_Cl_2_, MgSO_4_, Room Temperature, 12 h, Yield 95%; (c) 2,2′-(Ethylenedioxy)­bis­(ethylamine), CH_2_Cl_2_, MgSO_4_, Room Temperature, 12 h, Yield 97%

Following a previously reported microwave-assisted exfoliation method,^33,34^ MoS_2_–NS were obtained from MoS_2_ powder using a microwave (MW)-assisted method at 140 W for one hour, in the presence of *N*-Methyl-2-pyrrolidone (NMP), under a nitrogen atmosphere with high-speed stirring to ensure uniform mixing. The mixture was then centrifuged at 1500 rpm for 20 min to remove larger aggregates, and the resulting supernatant was characterized by UV–vis spectroscopy and used in subsequent reactions.

The UV–vis spectrum of the MoS_2_–NS exfoliated via microwave irradiation in *N*-methylpyrrolidone (NMP) as the solvent reveals distinctive spectral features, providing insights into the material’s optical properties (Figure 2). The spectrum displays two prominent humps at 614 and 675 nm, which are indicative of the typical excitonic transitions in MoS_2_–NS. These peaks are associated with the direct bandgap transitions of MoS_2_, with the peak at 614 nm often attributed to the A exciton and the one at 675 nm corresponding to the B exciton. The separation and intensity of these peaks are influenced by the exfoliation process, which enhances the optical response of MoS_2_ by increasing the number of monolayer or few-layer sheets. The use of NMP as a solvent during microwave irradiation helps to stabilize the exfoliated MoS_2_–NS, ensuring the high quality of the material and facilitating the observation of these distinct optical features. This UV–vis data are critical for understanding the electronic structure and the potential applications of MoS_2_ in optoelectronic devices. In agreement with the literature, C and D excitonic bands are observed at 460 and 340 nm, respectively.

On the basis of UV data other hand, three important parameters can be determined, namely the average length *L*, average concentration *C,* and the average number of layers *N,* using the following eqs 1–3:^24^

$$L\;(\mu m)=\frac{3.5\times \frac{{\mathrm{Ext}}_{B}}{{\mathrm{Ext}}_{350}}-0.14}{11.5-\frac{{\mathrm{Ext}}_{B}}{{\mathrm{Ext}}_{350}}}$$ 1

$$C=\frac{1}{l}{\mathrm{Ext}}_{350}/\varepsilon_{350}$$ 2

$$N=2.3\times 10^{36}\;e^{-54888{/}_{\lambda_{A}}}$$ 3

Based on the above equations, the *L* = 139 nm, *C* = 4.68 mg/mL, and *N* = 11 layers.

The morphology of the synthesized MoS_2_ nanosheets was examined by scanning electron microscopy with energy-dispersive X-ray spectroscopy (SEM/EDX) directly on the sensor’s carbon working electrode. As shown in Figure S12, which compares the bare SPCE with the MoS_2_-modified SPCE, the bare surface exhibits compact graphitic microplatelets. After MoS_2_ deposition, the electrode is uniformly covered by a flake-like nanosheet network with pronounced texturing and interflake voids. The related EDX spectra support this assignment: the bare SPCE shows only a dominant C (*K*α_1_) signal with a minor O (*K*α_1_) contribution, whereas the MoS_2_/SPCE displays clear Mo (*L*α_1_) and S (*K*α_1_) peaks in addition to C/O, confirming the presence of the dichalcogenide coating. To introduce carboxyl functionalities, a solution of MoS_2_ in NMP was reacted with MCA, exploiting the ability of chlorine atoms to fill sulfur vacancies in MoS_2._ ^29^ This reaction occurred in the presence of NaOH under ultrasonic conditions for 3 h, followed by an additional 12 h of stirring at room temperature (Scheme 2a). The MoS_2_–COOH sample was then obtained after purification by centrifugation. The conjugation of MoS_2_–COOH with the synthesized SBs was achieved by a coupling reaction between the carboxyl groups present in the NS and the free amino groups present in each SB. This reaction was performed in DMF at room temperature using *N*-(3-dimethylaminopropyl)-*N*′-ethylcarbodiimide hydrochloride (EDC·HCl) and hydroxybenzotriazole (HOBt) as coupling agents and in the presence of a catalytic amount of 4-dimethylaminopyridine (DMAP) (Scheme 2b).

**Scheme 2:**
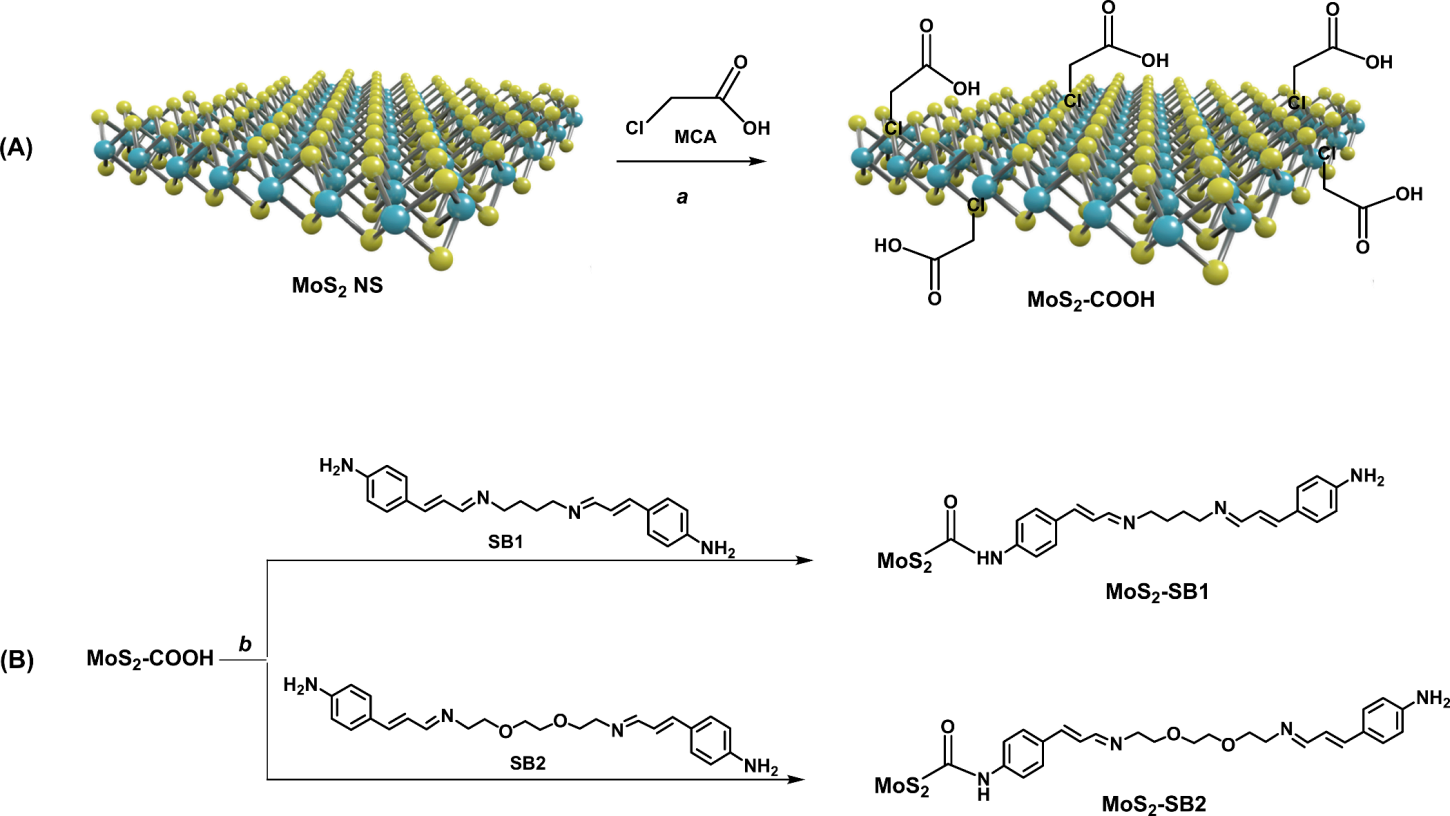
Synthesis of MoS_2_–SB1 and MoS_2_–SB2[Fn sch2-fn1]

a
*Reagents and Condition:* (a) Monocholoroacetic acid, NaOH, u.s 3 h, then r. t., overnight; (b) EDC·HCl, HOBt, SB1 or SB2, r.t. 48 h.

After purification by dialysis-bag technique, the MoS_2_-conjugated samples were characterized by Raman, UV–vis, and FTIR spectroscopy, while their thermal behavior was evaluated by TGA analyses.

Figure 3 shows the Raman characterization of the starting material synthesized by microwave irradiation (i.e., MoS_2_–NS) and the samples produced at various preparation steps as previously reported.^33^

**Figure 3:**
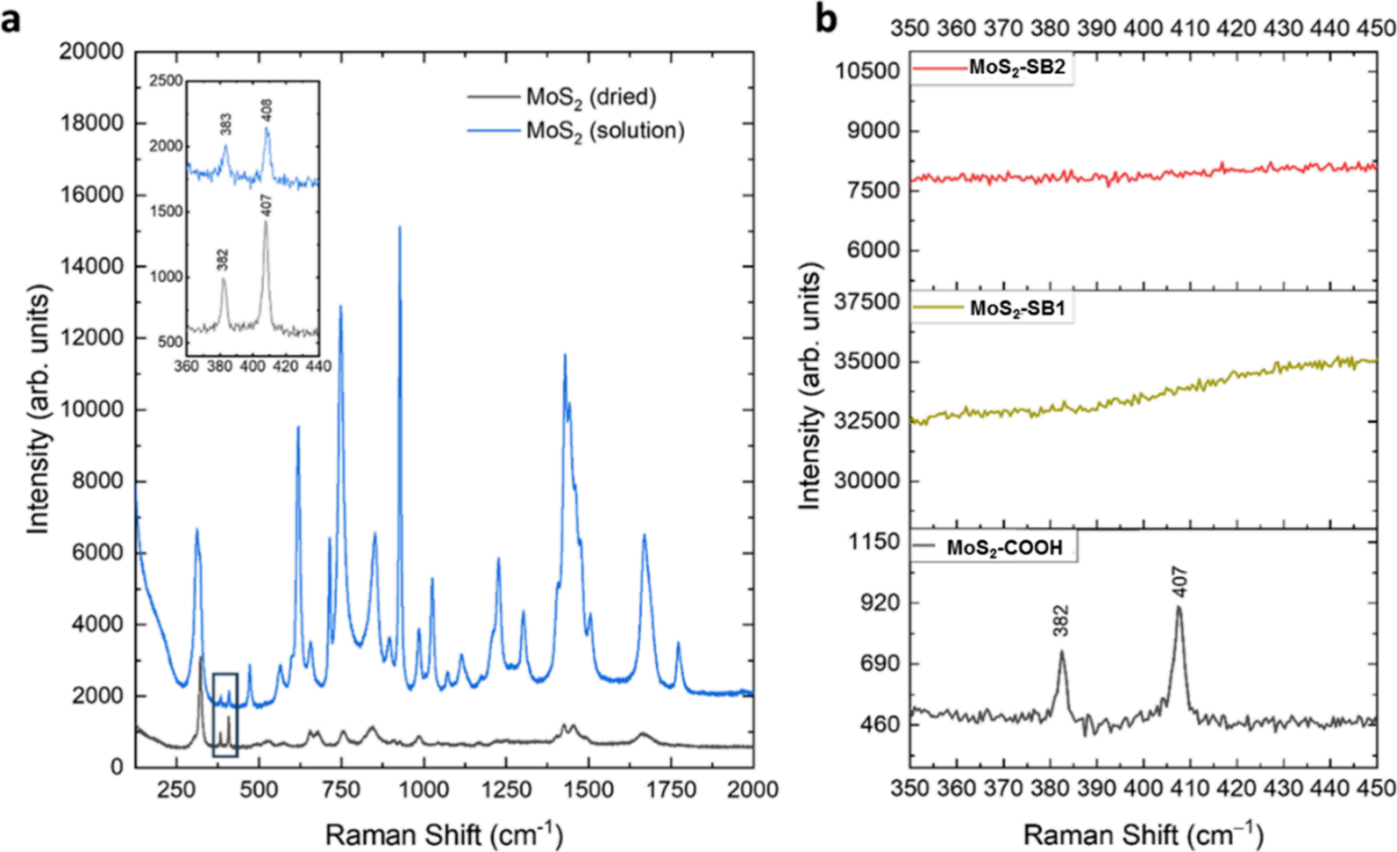
Raman spectra of (a) MoS_2_ dried and solution samples, and (b) samples of MoS_2_ treated with MCA (bottom) and combined with SB1 (middle) and SB2 (top). The inset in (a) focuses on the characteristic MoS_2_ spectral region.

The typical Raman spectrum of MoS_2_ features two main peaks: (i) an in-plane (*E*_2*g*_^1^) mode around 383 cm^–1^ involving the sulfur atoms vibrating in one direction and the molybdenum atom in the opposite direction - and (ii) an out-of-plane (*A*_1*g*_) mode of the sulfur atoms at 408 cm^–1.^ ^36,37^ As the layer thickness decreases toward a single layer, these two modes change with thickness, and the frequencies of the *E*_2*g*_^1^ and *A*_1*g*_ peaks serve as crucial indicators for determining the layer number of a MoS_2_ flake.

Figure 3a illustrates the data obtained for the exfoliated MoS_2_ samples, deposited via drop-casting on a CaF_2_ substrate, both in the liquid and “solid” (dried) states. Figure 3a shows the characteristic Raman modes of exfoliated MoS_2_, with shifts in *E*_2*g*_^1^ and *A*_1*g*_ peaks confirming the formation of quadrilayered sheets. In the Raman spectrum of MoS_2_ sample in solution (blue line), the typical Raman mode of the solvent is well visible and very intense. As expected, the solvent vibrational modes are less intense in the Raman spectrum of the dried samples (black line). In both spectra, the presence of the two characteristic disulfide peaks and the correct exfoliation of our MoS_2_ sample with the formation of a quadrilayer are confirmed.^38^ In fact, as reported in the inset of Figure 3a, in the case of the dried sample, the in-plane mode shifts up to almost 382 cm^–1^ and the out-of-plane mode shifts down to 407 cm^–1^.

Figure 3b shows the Raman signals obtained for the samples of MoS_2_ treated with MCA and combined with SB1 and SB2. In Figure 3b, while MCA-functionalized MoS_2_ (MoS_2_–COOH sample) retains the Raman signature, samples with Schiff bases exhibit strong fluorescence, hindering clear MoS_2_ signals. It is evident that the presence of MoS_2_ in the sample, combined with MCA (black line), is confirmed, retaining the characteristics of the original sample. However, in the samples with Schiff bases (red and dark yellow lines), there is a high fluorescence due to the use of the excitation laser at 532 nm that obscures any Raman signals due to MoS_2_.

UV–vis spectra of nanocomposites consisting of MoS_2_ and the two different Schiff bases reveal important insights into the interactions between MoS_2_ nanosheets and organic molecules. For the composite with SB1, the spectrum shows a peak at 350 nm, as shown in Figure 4a, upon integration of SB1 with MoS_2_, a red-shift of the π–π* transitions from 350 to 380 nm is observed, indicating electronic interaction. After integration with MoS_2_, the peak shifts to 380 nm, indicating a possible electronic interaction between MoS_2_ and the dianiline derivative, which alters the electronic structure of the organic component. Furthermore, the two peaks at 614 and 675 nm are characteristic of exfoliated MoS_2_, indicating the presence of MoS_2_ nanosheets in the composite. These peaks are attributed to the intrinsic optical transitions of MoS_2_, particularly the A and B excitons related to its direct bandgap properties. Similarly, in Figure 4b, MoS_2_–SB2 displays new bands at 368 and 443 nm, reflecting changes in the electronic environment due to Schiff base incorporation. For the second system with SB2, the UV–vis spectrum of the organic compound shows two peaks at 320 and 350 nm, corresponding to the π-π* transitions of the conjugated Schiff base molecules (Figure 4b). In combination with MoS_2_, the peaks shift slightly to 328 nm, 368 and 443 nm, indicating an interaction effect similar to that observed in the previous system, in which MoS_2_ influences the electronic transitions of organic molecules. The presence of the two MoS_2_ peaks at 614 and 675 nm further confirms the interaction of MoS_2_ with the organic compound, as the optical transitions of MoS_2_ are conserved in the composite. These shifts of the absorption peaks suggest a significant change in the electronic properties of MoS_2_ and the organic molecules due to their interactions, which could lead to the formation of charge transfer complexes or altered electronic states.

**Figure 4:**
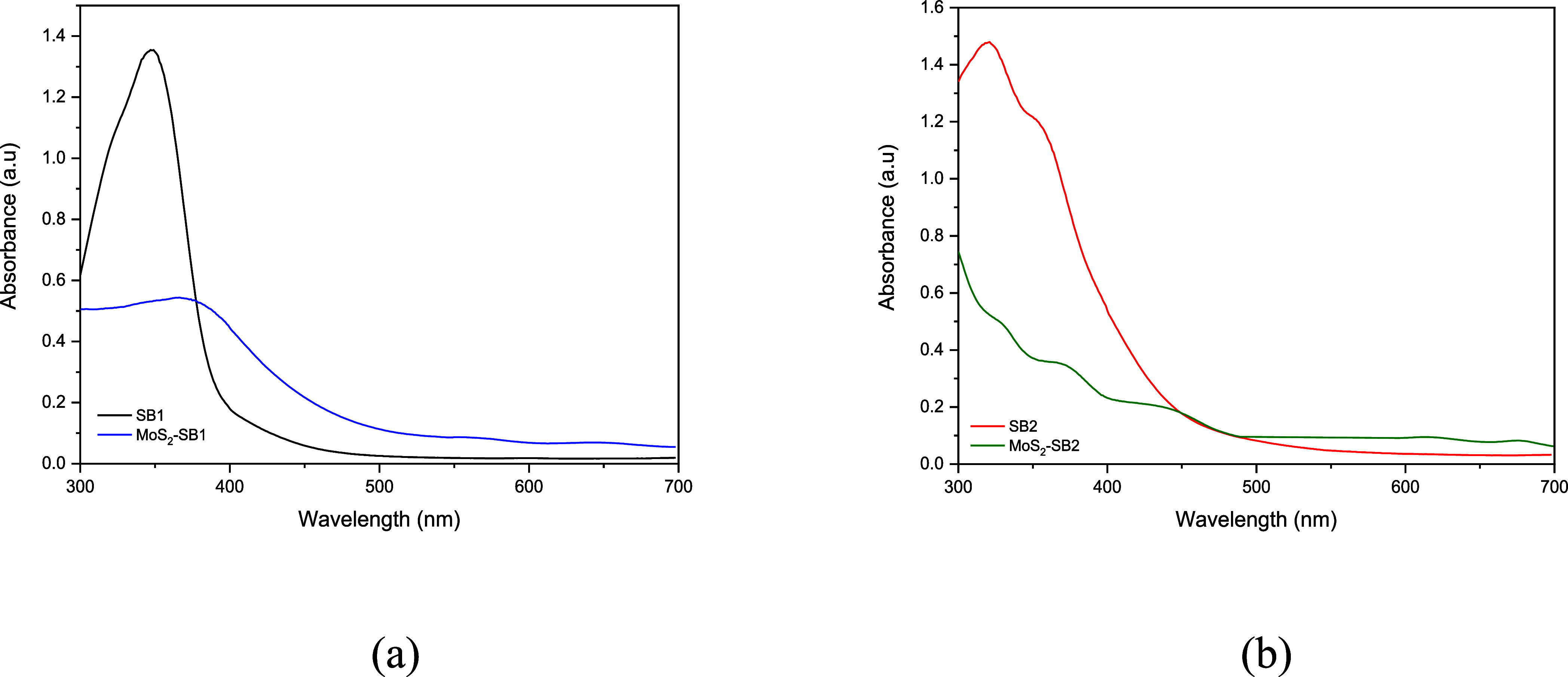
(a) UV–vis absorption spectrum of SB1 and MoS_2_–SB1; (b) UV–vis absorption spectrum of SB2 and MoS_2_–SB2.

Figure 5 presents the FTIR spectra of the conjugated samples MoS_2_–SB1 and MoS_2_–SB2, along with those of the starting Schiff bases (SB1 and SB2). The spectra confirm the presence of characteristic functional groups in SB1 and SB2, as well as their effective bonding to MoS_2_, evidenced by shifts in the imine, amine, and Mo–S related regions. The FTIR spectrum of sample SB1 shows the stretching of the aromatic C–H of the anilines (about 3000 cm-^1^); the carbon–carbon bonds within the aromatic rings give rise to bands in the 1500 cm^–1^ region. These are generally medium bands associated with in-plane bending of aromatic rings. The imine groups (−C=N−) of the butane-1,4-diylbis­(azaneylidene) moiety of the molecule produce a stretching vibration around 1600 cm^–1^, characteristic of the C=N bond, while the conjugated C=C double bonds in the propene-bonded parts of the molecule exhibit stretching frequencies in the 1600 cm^–1^ range. Aniline groups can exhibit NH_2_ bending modes, which generally appear near 1600 cm^–1^; C–N stretching vibrations, particularly those of nitrogen atoms in imine groups or amine bonds, show peaks around 1200–1300 cm^–1^. The FTIR spectrum of MoS_2_–SB1 shows the presence of new peaks associated with the MoS_2_ material itself, particularly in the 400–600 cm^–1^ range, corresponding to Mo–S vibrations. Reaction with monochloroacetic acid introduces new peaks due to the formation of ester groups or carboxylic functionalities, typically observed around 1700 cm^–1^ (for C=O stretching) and 1300 cm_–_^1^ (for C–O stretching). Shifts in the amine or imine regions are also recorded, reflecting interactions between the molecular compound and MoS_2_ nanosheets, indicating successful functionalization. The FTIR spectrum of sample SB2 shows the stretching of the C–H hybridized sp^2^ of aromatic systems around 3000 cm^–1^, and the C–C stretching vibrations around 1500 cm^–1^. Around 1600 cm^–1^ there are strong absorption bands due to stretching of the (−C=N−), typical of C=N stretching in conjugated systems. The (−O−) ether bonds at positions 7 and 10 show characteristic absorptions of C–O stretching around 1100 cm^–1^. After the functionalization reaction, again there are new peaks associated with the MoS_2_ material itself, particularly in the range of 400–600 cm^–1^, corresponding to Mo–S vibrations. The overall FTIR spectrum confirms the effective chemical bond formation and structural changes of the original compound after modification with MoS_2_.

**Figure 5:**
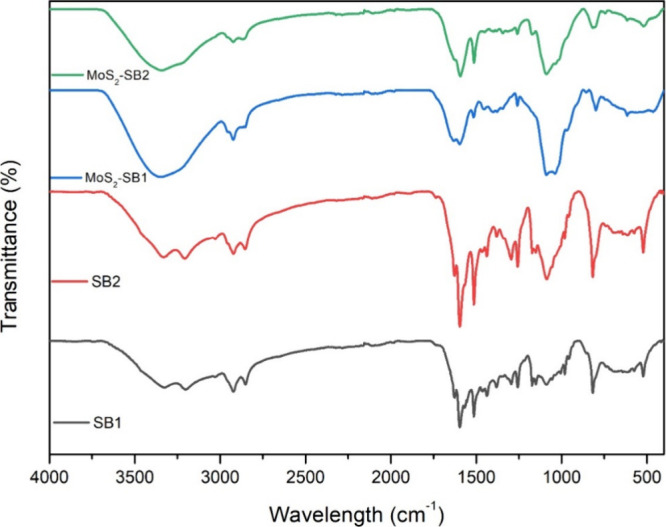
FT-IR spectrum of SB1, SB2, MoS_2_–SB1 and MoS_2_–SB2.

Thermogravimetric analysis (TGA) (Figure 6) first highlights the reference behavior of the unfunctionalized MoS_2_ nanosheet (MoS_2_–NS), which shows minimal mass loss over 100–700 °C and a high residual mass (∼90% at 700 °C). This slight, nearly linear decrease reflects the intrinsic thermostability of the inorganic MoS_2_ nanosheet under an inert atmosphere. In comparison, the MoS_2_–COOH nanosheet exhibits an intermediate thermal evolution: after a short induction region (<∼140 °C), a gradual and continuous mass loss is observed between ∼200 and 600 °C, attributable to the decomposition of organic fragments introduced by the treatment (cleavage/decarboxylation of −COOH groups and the −CH_2_– bridge). At 700 °C, the sample typically retains ∼50% residual mass, consistent with a moderate organic loading and the effective introduction of carboxyl groups onto the nanosheet. The thermogravimetric analysis profiles of MoS_2_–SB1 and MoS_2_–SB2 show a very similar thermal behavior. Both composites exhibit excellent thermal stability, remaining stable up to 140 °C with no significant weight loss in this temperature range. This suggests that both nanocomposites have good resistance to thermal degradation at low temperatures, likely due to the stability of the MoS_2_ matrix and the functional groups attached to it. After 140 °C, both composites show steady and continuous weight loss, indicating the gradual decomposition or volatilization of the organic components. This implies that the organic ligands bound to the MoS_2_ nanosheets are the primary sources of weight loss at higher temperatures. Both MoS_2_–SB1 and MoS_2_–SB2 retain a small residual mass at 700 °C of 10 and 9%, respectively.

**Figure 6:**
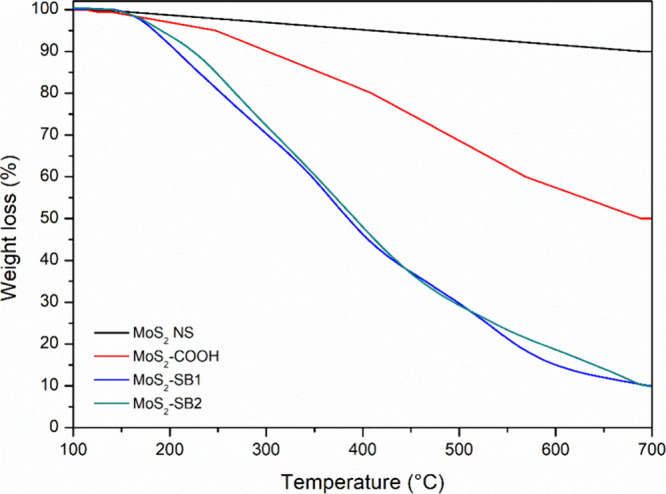
TGA profiles of MoS_2_–SB1 and MoS_2_–SB2.

Overall, both characterization results provide strong evidence for the successful formation of MoS_2_–SB nanocomposites, with retained structural integrity and functional properties.

Zeta potential measurements further substantiate successful surface modification of the exfoliated MoS_2_ nanosheets. MoS_2_–NS exhibits −40.3 mV, while MoS_2_–COOH shifts to −32.5 mV; the ligand-bearing samples MoS_2_@SB1 and MoS_2_@SB2 display −21.8 mV and −24.4 mV, respectively. This systematic decrease in the magnitude of the negative ζ-potential is consistent with progressive organic coverage and is in agreement with literature reports for −CH_2_COOH-functionalized MoS_2_ nanosheets,^39^ where ζ becomes less negative after covalent grafting.

### 3.2 Electrochemical Studies

The electrochemical behavior of carbon-based SPEs modified with MoS_2_–SB1 and MoS_2_–SB2 composites via drop-casting was initially evaluated using cyclic voltammetry (CV) and electrochemical impedance spectroscopy (EIS), and the results compared to the responses of the unmodified (bare) SPCE (Figure 7). The CV analyses shown in Figure 7a were performed by sweeping the potential from −0.4 V to +0.8 V at a scan rate of 50 mV/s, using a 5 mM K_3_[Fe­(CN)_6_] solution in PBS as the electrolyte. All tested electrodes displayed the characteristic redox peaks of the probe, with MoS_2_/SPCE and MoS_2_–SB2/SPCE showing the largest faradaic peaks, while bare/SPCE exhibited the lowest currents. SB-modified electrodes also showed a smaller peak-to-peak separation (Δ*E*_p_) than bare/SPCE and MoS_2_/SPCE. This enhancement is attributed to improved charge transfer and increased conductivity due to Schiff base integration. Although MoS_2_–MCA can display relatively high peaks in the CV probe, that response mainly reflects effective area, double-layer charging, and probe-dependent electron-transfer kinetics, and therefore does not predict stripping performance. By contrast, MoS_2_–SB forms a covalently defined interfacial layer with donor sites that stabilizes the surface and promotes interfacial accumulation of the target ions during the deposition step, the factors that ultimately govern the analytical signal under SWASV. Accordingly, SB functionalization remains warranted even when the CV-probe peak of MoS_2_–SB is not the largest among the MoS_2_-based electrodes.

**Figure 7:**
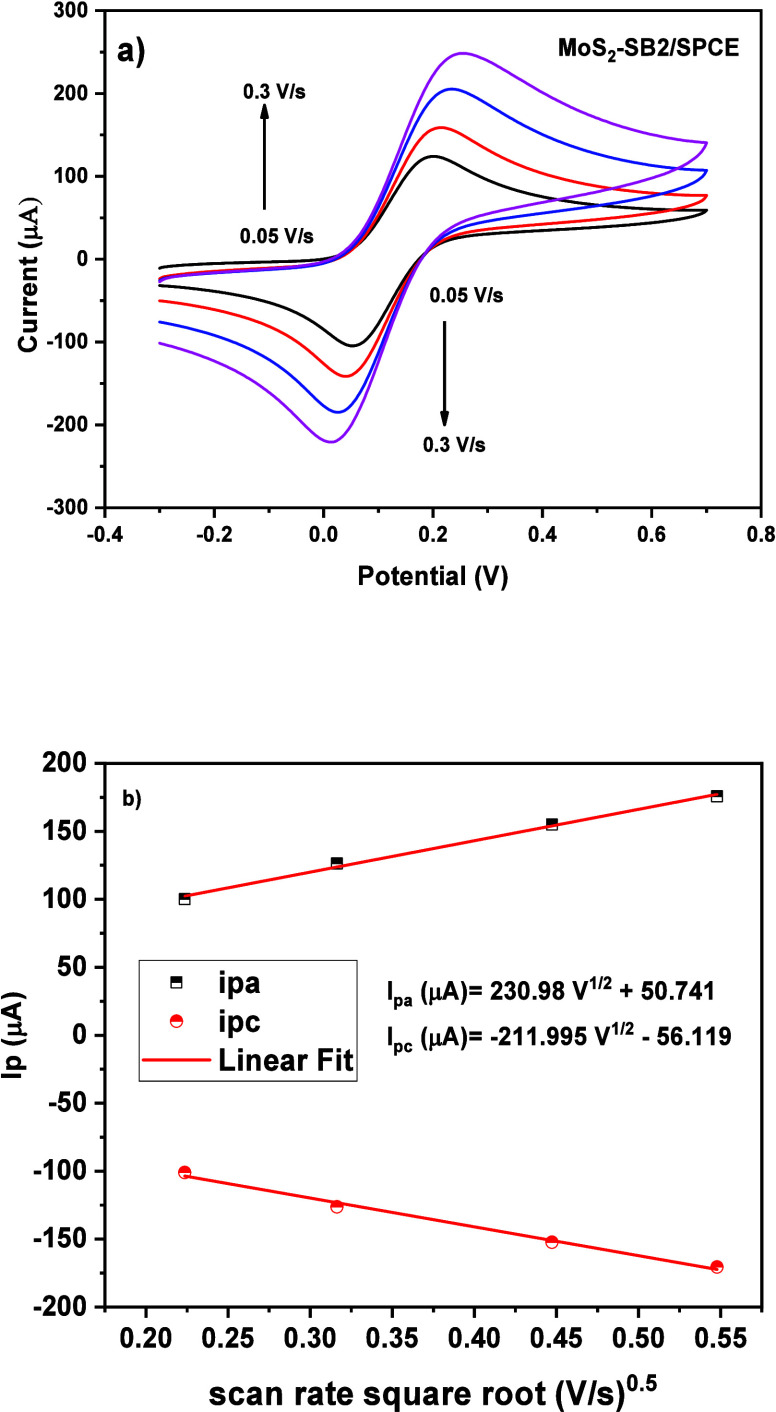
(a) Scan rate study on the MoS_2_–SB2/SPCE and (b) the variation of scan rate square root Vs current.

The scan rate variation from 0.05 V/s to 0.3 V/s was also performed on MoS_2_–SB2/SPCE in 10 mM ferrocyanide solution. As presented in Figure 7a, increasing the scan rate increases both anodic and cathodic currents. The linear relation between the scan rate and the square root is shown in Figure 7b, proving that on the MoS_2_–SB2/SPCE sensor the electrochemical reaction occurs via a diffusion-controlled mechanism. Herein, the active surface area is found using the following eq 4:

$$I_{\mathrm{pa}}(A)=2.69\times 10^{5}An^{3/2}CD^{1/2}\nu^{1/2}$$ 4

where ν is the scan rate expressed V/s, *D* is the diffusion coefficient (7.6 × 10^–6^ cm^2^ s^–1^),^27,28^ *A* is the electroactive surface area expressed in cm^2^, *n* = 1 is the number of electrons involved in the redox reaction of Fe­[(CN)_6_]^3–/4–^, and C is the concentration of Fe­[(CN)_6_]^3–/4–^ expressed in mol/cm^3^. From the experimental data, we find an EASA = 0.031 cm^2^.

These findings are in perfect agreement with the Nyquist plots achieved by the EIS study that were studied under applied potential 0.112 V (see Figure 8b). The equivalent circuit is shown in the inset Figure 8b, which is obtained through data fitting via the Nova 2.1 software; experimental fit overlays are provided in Figures S6–S10. The main components are observed: the charge transfer resistance (*R*_CT_) and the electrolyte-electrode surface resistance (*R*_s_), whose fitted values are summarized in Table 1. Quantitatively, *R*_ct_ decreases from 2449 Ω (bare/SPCE) and 2406 Ω (MoS_2_/SPCE) to 579 Ω (MoS_2_–SB1/SPCE) and 442 Ω (MoS_2_–SB2/SPCE) (Table 1), indicating that electron transfer on the modified surfaces is enhanced compared with MoS_2_/SPCE and bare/SPCE. Conversely, Rs reflects the electrode/electrolyte interfacial kinetics and remains in the same order of magnitude across all electrodes (Table 1). These results highlight a key advantage of the proposed modified electrochemical platform.

**Figure 8:**
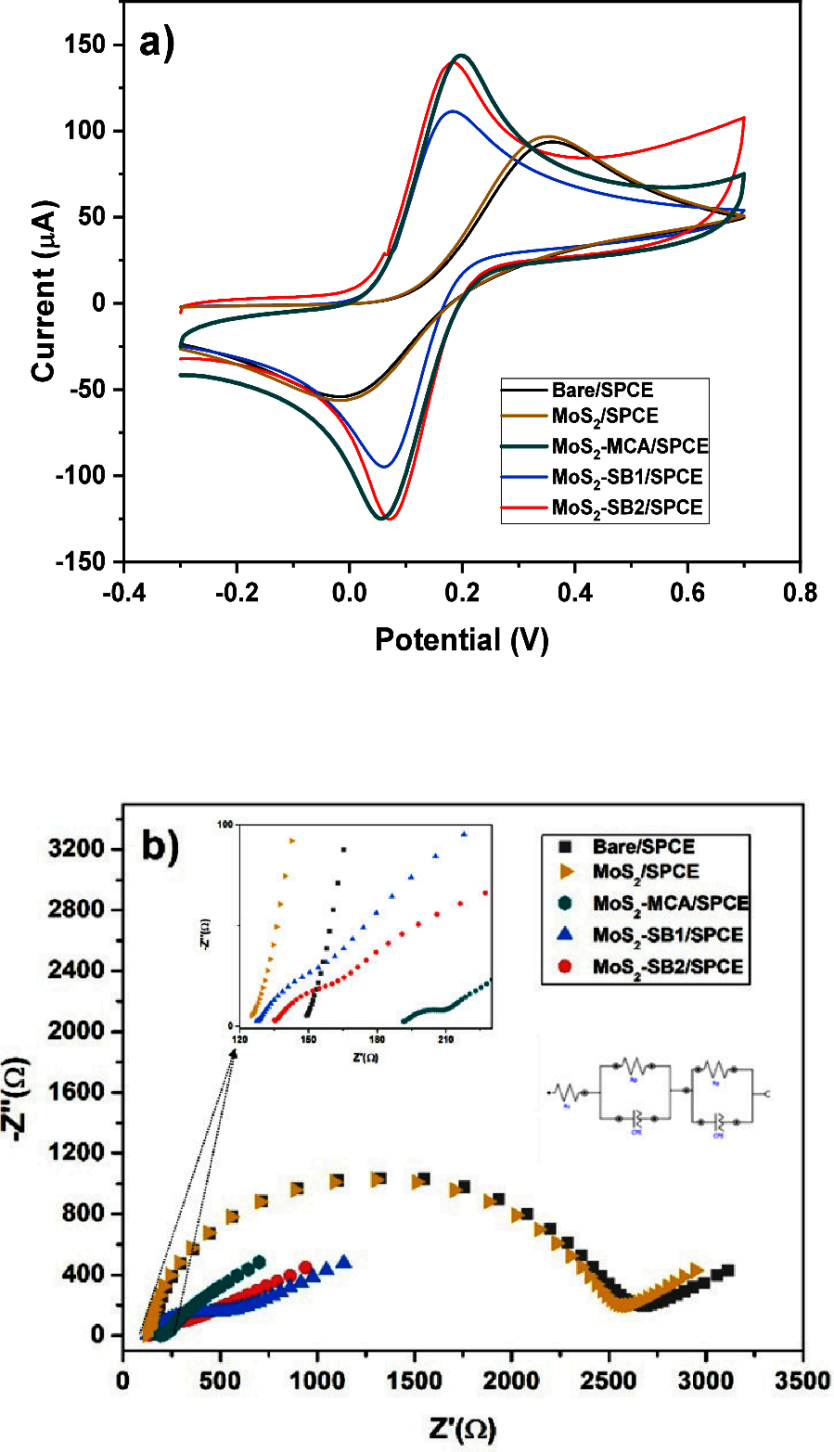
(a) CV curves and (b) Nyquist plots of the modified and unmodified SPCE electrodes in 10 mM of ferrocyanide solution. The inset magnifies the high-frequency region (Z′ ≈ 120–230 Ω), highlighting the small semicircle observed for the functionalized electrodes.

**Table 1:** Randles Parameters Obtained from Fitting Using Nova 2.1 Software

**Randles parameters**					
**sensors**	*R*_CT_ (Ω)	*R*_s_ (Ω)	CPE-*t*	CPE-*n*	W
bare/SPCE	2449	139	1.07 μMho·s^n^	0.903	100 μMho·s^1/2^
MoS_2_/SPCE	2406	116	1.07 μMho·s^n^	0.903	100 μMho·s^1/2^
MoS2-MCA/SPCE	2192	113	3.90 μMho·s^n^	0.814	100 μMho·s^1/2^
MoS_2_–SB1/SPCE	579	131	3.30 μMho·s^n^	0.644	100 μMho·s^1/2^
MoS_2_–SB2/SPCE	442	134	8.08 μMho·s^n^	0.508	100 μMho·s^1/2^

The optimization of the electrolyte type and pH was carried out at room temperature using the bare SPCE for the detection of various heavy metals. Figure 9a presents the results obtained in phosphate buffer solutions (PBS) across a pH range of 4.0–8.0, with a fixed heavy metal concentration of 10 μM. The highest current responses for Pb^2+^, Cd^2+^, and Cu^2+^ were observed under acidic conditions. A similar trend was noted in acetate buffer solution (ABS), though with overall lower current responses (Figure 9b). Although Pb^2+^ was the main target analyte, other heavy metal ions (Cd^2+^, Cu^2+^, Zn^2+^, Ni^2+^) were also considered during the pH optimization to evaluate possible interferences, since these cations often coexist in real samples. The pH strongly influences Pb^2+^ speciation, solubility, and deposition efficiency, with acidic conditions favoring higher stripping currents and neutral/alkaline media leading to decreased responses due to hydrolysis or precipitation. Based on these findings, PBS at pH 4 was selected for further experiments, as it provided the most favorable electrochemical response.

**Figure 9:**
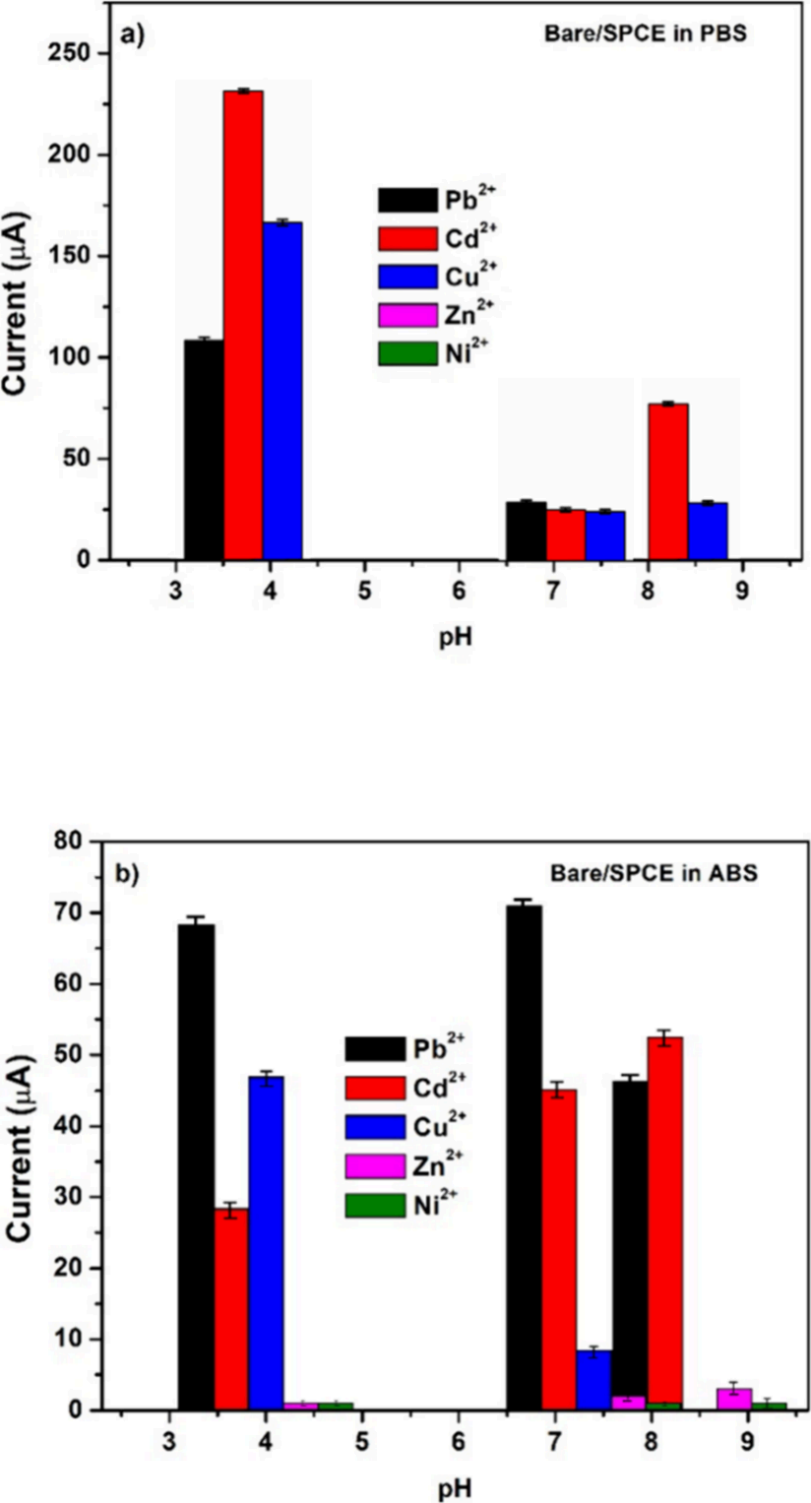
pH optimization study in (a) phosphate buffer solution (PBS) and (b) acetate buffer solution (ABS) on bare/SPCE at 10 μM of different heavy metals M^2+^.

Under the optimized conditions, the electroanalytical behavior of the bare and modified electrodes toward Pb^2+^ was systematically evaluated. Molecular dynamics (MD) and density functional theory (DFT) simulations previously indicated that SB2 interacts more strongly with Pb^2+^ than SB1 [28], suggesting a possible performance enhancement. Figure 10a–d show the SWASV responses of the bare SPCE, MoS_2_/SPCE, MoS_2_–SB1/SPCE, and MoS_2_–SB2/SPCE in PBS (pH 4.0) over the concentration range 1–10 μM Pb^2+^. The bare electrode displayed only a weak stripping signal (Figure 10a), while incorporation of MoS_2_ significantly increased the current response (Figure 10b), confirming the electrocatalytic contribution of the nanomaterial. Functionalization with SB1 further improved sensitivity (Figure 10c), but the best performance was achieved with SB2 (Figure 10d), in line with the predicted stronger affinity of this ligand for Pb^2+^.

**Figure 10:**
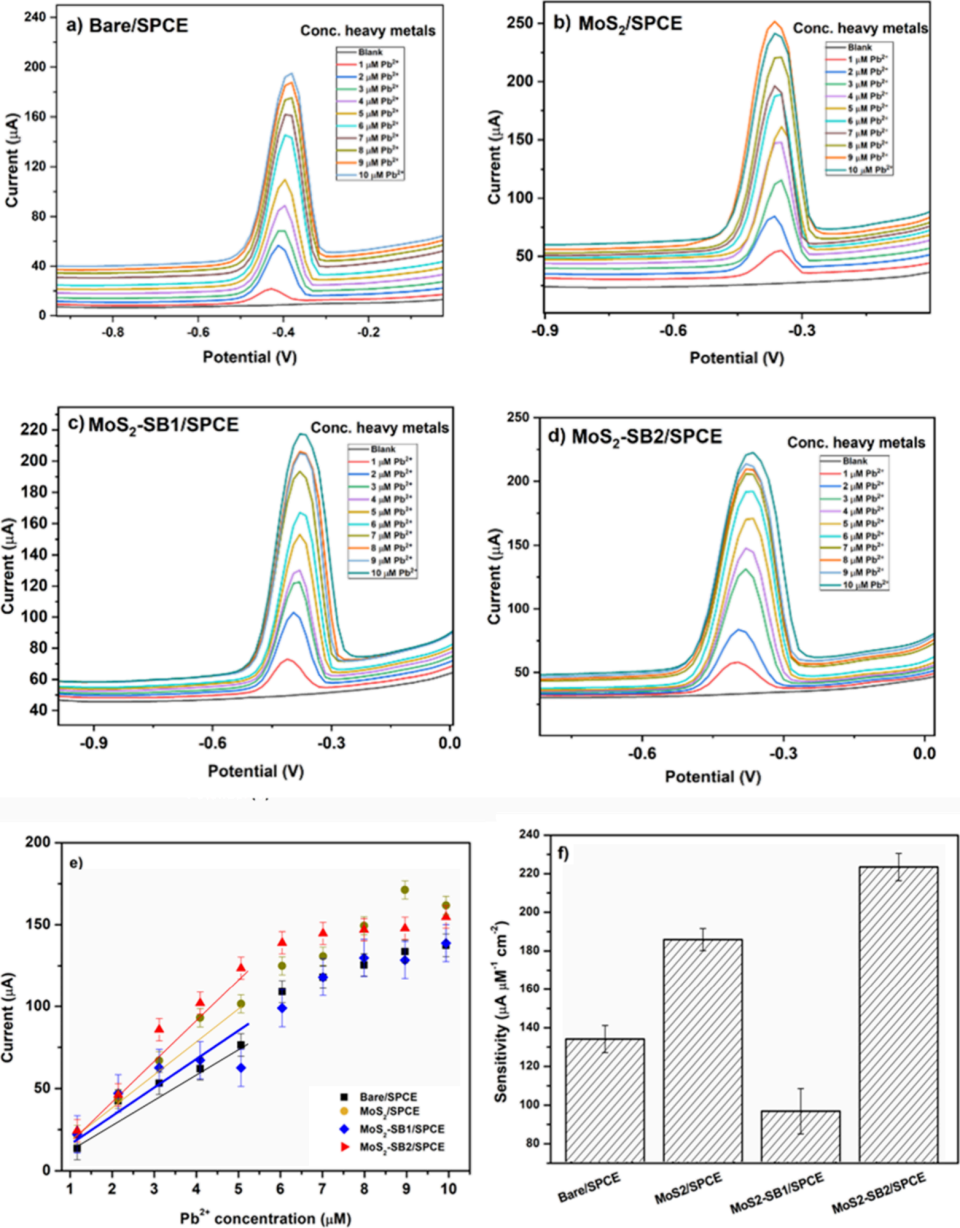
SWASV curves of (a) bare/SPCE, (b) MoS_2_,/SPCE, (c) on MoS_2_–SB1/SPCE, (d) MoS_2_–SB2/SPCE, (e) calibration curves of bare and modified electrodes reported as mean ± SD (*n* = 3 independently fabricated electrodes; three technical replicates each) and (f) sensitivity of bare and modified electrodes in the linear Pb^2+^ concentration range of 1 μM −5 μM. RSD% **=** bare 7.4, MoS_2_ 6.0, MoS_2_–SB1 12.3, MoS_2_–SB2 7.4.

The regression analysis of the calibration plots (Table 2) confirmed good linearity for Bare/SPCE, MoS_2_/SPCE, and MoS_2_–SB2/SPCE (*R* ^2^ > 0.94), whereas MoS_2_–SB1/SPCE exhibited a lower correlation coefficient (*R* ^2^ = 0.745), likely due to increased variability in electrode response. The corresponding calibration curves (Figure 10e), reported as mean ± SD (*n* = 3 independently fabricated electrodes; three technical replicates each), revealed two distinct linear regimes (1–5 μM and 6–10 μM). Sensitivity values derived from these ranges are summarized in Figure 10f. Among the tested platforms, MoS_2_–SB2/SPCE demonstrated the highest sensitivity (220 μA μM^–1^ cm^–2^ in the 1–5 μM range), significantly outperforming MoS_2_–SB1/SPCE (87 μA μM^–1^ cm^–2^). The limit of detection (LoD) was also lowest for MoS_2_–SB2/SPCE (0.267 μM), confirming the beneficial synergistic effect of MoS_2_ and the SB2 ligand. Overall, the proposed architecture achieves a very steep calibration slope on disposable SPCEs (220 μA μM^–1^ cm^–2^) with good interdevice reproducibility (RSD = 7.4%), although it maintains a relatively narrow primary linear range (1–5 μM) and a LoD that does not reach the ultratrace levels reported for more elaborate systems. In comparison with recent literature, the MoS_2_–SB2/SPCE sensor offers superior sensitivity and a lower LoD, justifying its selection for further studies and highlighting the effectiveness of this functionalization strategy for heavy-metal detection. Table 2 summarizes the performance of all four electrodes, while Table 3 positions our platform against representative Pb^2+^ sensors. Notably, the area-normalized sensitivity of our sensor (∼220 μA μM^–1^ cm^–2^) is approximately 1 order of magnitude higher than ref 29, ∼160-fold higher than ref 33 and ∼13-fold higher than ref 38. Thus, while ultratrace LoDs can be achieved with more sophisticated architectures, our Pb^2+^-chelating, noble-metal-free, disposable platform delivers substantially enhanced sensitivity, which is advantageous for rapid on-site quantification with minimal preconcentration.

**Table 2:** Linear Regression Equations and Correlation Coefficients (*R* ^2^) Obtained from Calibration Plots for Pb^2+^ Detection Using Bare and Modified SPCEs

**electrodes**	**calibration equation (μA)**	***R*** ^**2**^
bare/SPCE	*I* = (1.887 ± 7.3893) + (15.8036 ± 2.2280) *C*_Pb_	0.9373
MoS_2_/SPCE	*I* = (−1.398 ± 6.0195) + (22.625 ± 1.815) *C*_Pb_	0.98106
MoS_2_–SB1/SPCE	*I* = (19.375 ± 12.287) + (10.968 ± 3.705) *C*_Pb_	0.7450
MoS_2_–SB2/SPCE	*I* = (−4.265 ± 7.3881) + (27.543 ± 2.2276) *C*_Pb_	0.98075

**Table 3:** Comparative Table of the Sensing Parameters

**electrodes**	**sensitivity (μA μM** ^**–1**^ **cm** ^**–2**^ **)**	**LoD (μM)**	**linear range (μM)**	**ref**
AuNps-L^1^/SPCE	56	0.298	[0.24;1,6]	^15^
ZnO–Nafion/GCE	0.113	0.09	0.1–2.0	^17^
Bi–Fe–carbon xerogel/GCE	8.44	0,000033		^18^
Bio-ZnO-NPs/CPE	57.2	0.41	3–9	^19^
Fe_3_O_4_@Schiff base Network1		0.0009		^38^
graphite/cork	0.8	0.3	[1;25]	^40^
SPCE/LPE/BIONPs/PANI	65	0.002	[0.45; 5.43]	^41^
SPCE/OPE/BiONPs/PANI	27	0.013	[0;4.03]	^41^
CoFe_2_O_4_@C- 600/GCE	22.22	0.0043	[0.001–60]	^42^
AuCuNC@N-GQD	1.39	0,000001	[0.000001–1000]	^43^
Co_3_O_4_–NC/SPCE	16.73	0,0041	[0.01–8.38]	^44^
bare/SPCE	126	1.40	[1;5]	this work
MoS_2_/SPCE	180	0.79	[1;5]	this work
MoS_2_–SB1/SPCE	87	3.36	[1;5]	this work
MoS_2_–SB2/SPCE	220	0.26	[1;5]	this work

#### 3.2.1 Electroanalytical Performance toward the Simultaneous Determination of Pb^2+^and Cd^2+^

Considering the improved electrochemical response of the bare electrode in phosphate buffer solutions (PBS) across a pH range of 4.0–8.0 toward Cd^2+^ ions, using a fixed concentration of 10 μM, we aimed to evaluate the sensitivity of the modified electrode toward this ion as well. Moreover, given the similarity in response, we also investigated potential interference effects in the presence of both Cd^2+^ and Pb^2+^ ions. Electrochemical measurements using SWASV revealed that the MoS_2_–SB2-modified electrode generated a distinct stripping peak for Cd^2+^ at approximately −0.7 V (Figure 11a). Although the current response for Cd^2+^ was lower and the detection limit higher than that for Pb^2+^, the signal remained stable and reproducible, confirming the electrode’s capability to detect both ions. To further evaluate its performance, simultaneous detection of Pb^2+^ and Cd^2+^ was carried out in PBS buffer (pH 4.0) with both analytes present at varying concentrations (0.5–6 μM). As shown in Figure 11b, two well-resolved anodic peaks corresponding to Pb^2+^ and Cd^2+^ were observed without signal overlap, demonstrating the sensor’s excellent selectivity and resolution for dual-ion detection. The current for Pb^2+^ reached nearly 175 nA at 6 μM, while Cd^2+^ showed a maximum current around 115 nA at the same concentration, reflecting the sensor’s higher sensitivity toward Pb^2+^.

**Figure 11:**
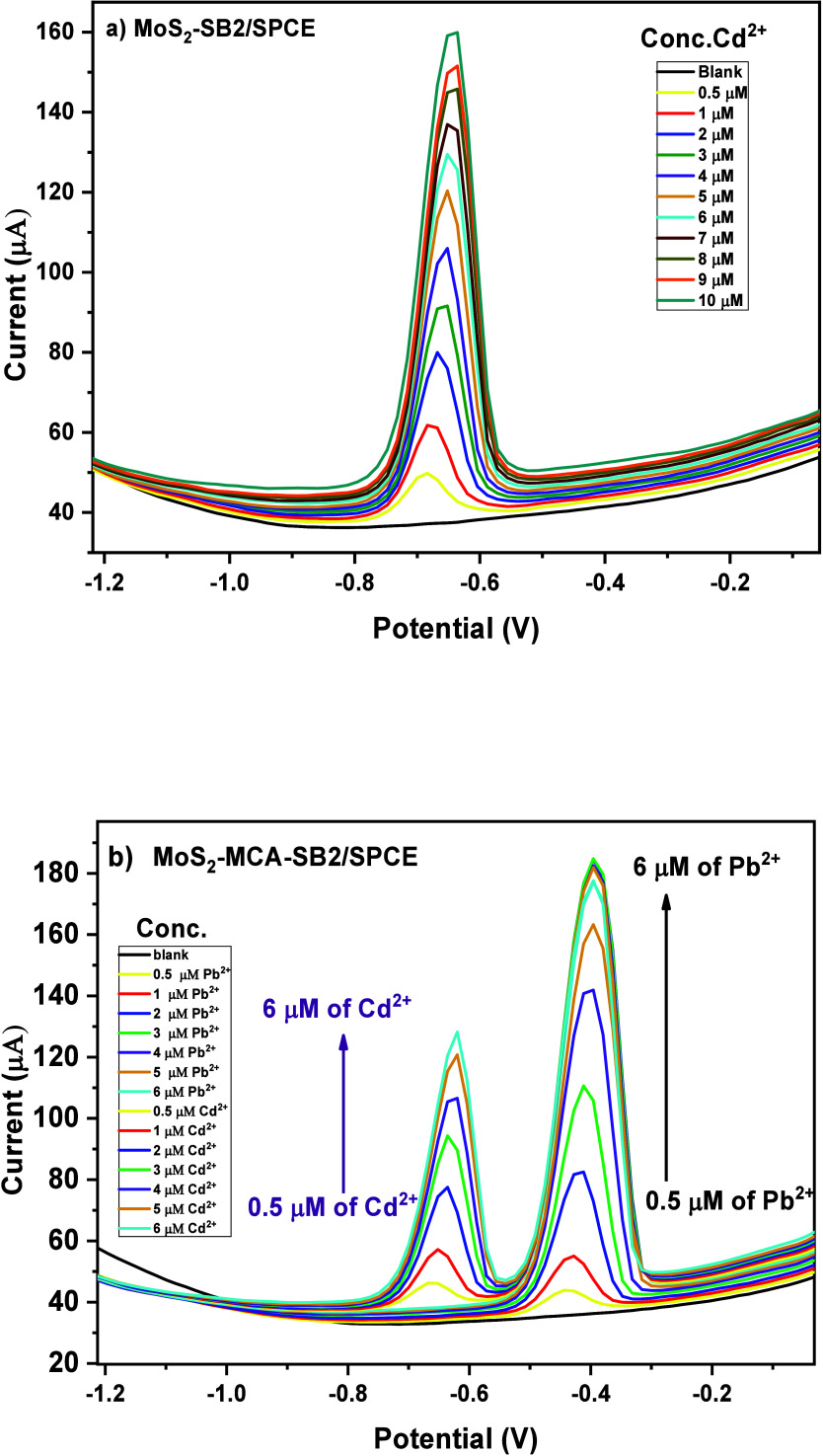
SWASV curves (a) of Cd^2+^ at different concentrations (b) the simultaneous determination of Pb^2+^ and Cd^2+^ on MoS_2_–SB2/SPCE.

Mechanistically, detection is governed by chelation between the Schiff base (SB2) and the target cations: the azomethine nitrogen serves as an electron-donating site that coordinates metal ions, enabling surface preconcentration prior to the stripping step. The stronger electronic interaction and binding affinity of SB2 for Pb^2+^, consistent with our MD/DFT indications, lead to higher surface coverage and more efficient electron transfer than for Cd^2+^, thereby producing a larger oxidation current for Pb^2+^ in simultaneous detection. In addition, the intrinsically slower redox kinetics of Cd^2+^ further contribute to its lower peak current relative to Pb^2+^. In our configuration, the primary sensitivity increases stems from the MoS_2_–SB overlayer; therefore, using a more exotic substrate (e.g., carbon-nanofiber or graphene SPEs) would likely trade additional area for higher capacitive background and peak broadening in SWASV, without a clear benefit over the simpler and more reproducible SPCE platform adopted here.

#### 3.2.2 Selectivity, Reproducibility, and Repeatability Studies

Three essential performance evaluations selectivity, reproducibility, and repeatability were carried out on the MoS_2_–SB2-modified electrode under identical experimental conditions. As illustrated in Figure 12a, the selectivity test was performed in PBS buffer (pH 4.0), where SWASV measurements were taken for each heavy metal ion at a concentration of 4 μM. Distinct anodic peaks appeared at −0.4 V for Pb^2+^, −0.6 V for Cd^2+^, and −0.03 V for Cu^2+^, demonstrating the sensor’s ability to selectively distinguish these ions without overlapping signals. In contrast, no peaks were observed for Zn^2+^ and Ni^2+^, indicating that the MoS_2_–SB2 nanocomposite shows minimal or no interaction with these metals under the given conditions. To evaluate reproducibility, three separate SPCEs were modified with the same amount of MoS_2_–SB2 and tested via SWASV in a 4 μM Pb^2+^ solution. The highly consistent current responses (Figure 12b) confirmed excellent fabrication reproducibility. Finally, repeatability was examined by conducting three consecutive measurements using the same modified electrode under unchanged conditions. Since the electrode is designed for single use, repeatability on the same device is not applicable. As illustrated in Figure 12c, performing consecutive measurements with the same MoS_2_–SB2/SPCE in 4 μM Pb^2+^ causes a progressive decrease in current. This decline arises from surface passivation, most likely due to the strong and irreversible chelation of Pb^2+^ ions by the SB2 ligand, which saturates the available binding sites. Such behavior confirms the disposable nature of the sensor: each electrode is intended for a single measurement, ensuring reliable selectivity while minimizing fouling and carry-over effects.

**Figure 12:**
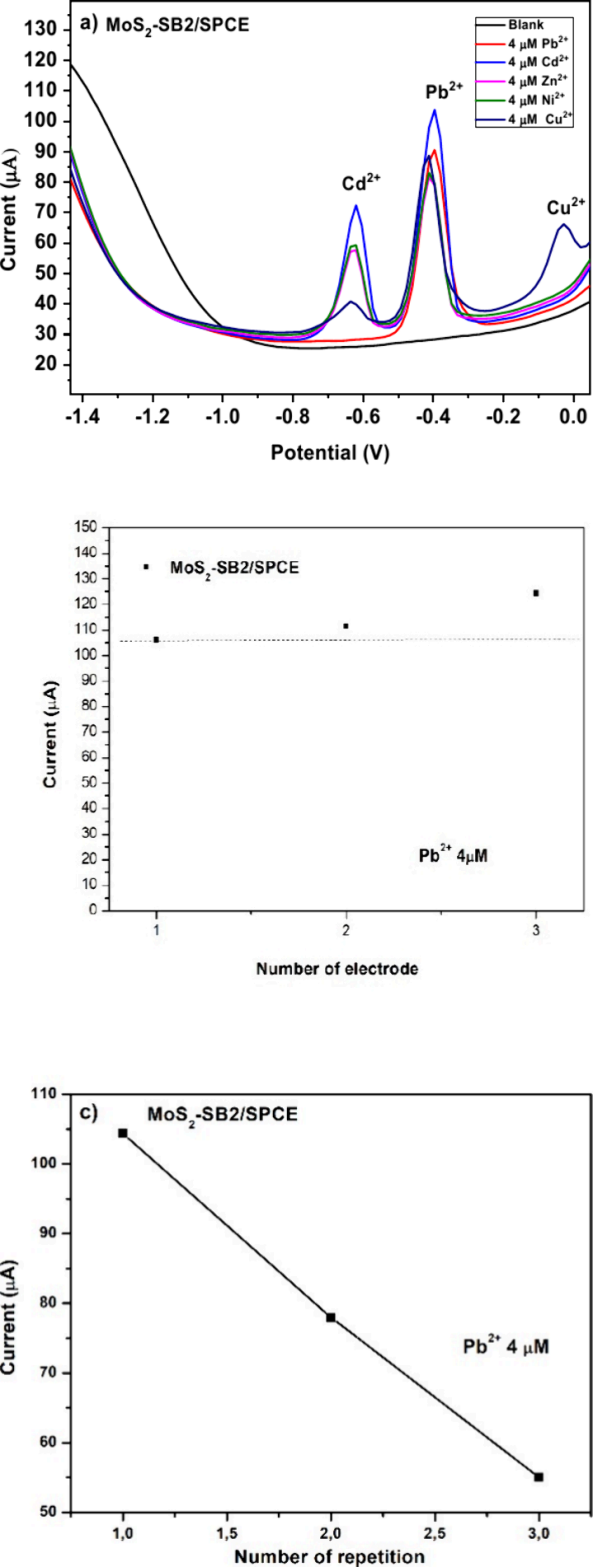
(a) Selectivity, (b) reproducibility, and (c) repeatability studies on MoS_2_–SB2/SPCE.

#### 3.2.3 Real Sample Analyses

The practical applicability of the MoS_2_–SB2/SPCE-modified electrode for the simultaneous detection of Pb^2+^ and Cd^2+^ ions was evaluated in a real matrix, specifically tap water with an initial pH of 7. Prior to the SWASV measurements, the pH was adjusted to 4.0 by addition of 2 M HCl, in accordance with the optimized conditions established in PBS. Cd^2+^ was deliberately included as a coanalyte to emulate realistic cocontamination scenarios and to stress-test selectivity and potential mutual interference under matrix effects. As shown in Figure 13a, both Pb^2+^ and Cd^2+^ produced well-defined and separate anodic stripping peaks at −0.4 V and −0.7 V, respectively, confirming that peak resolution is preserved in a complex matrix and enabling accurate quantification of Pb^2+^ in the presence of Cd^2+^. The sensitivity of the sensor toward Pb^2+^ in tap water was slightly reduced compared to PBS, with a calculated value of 126 μA μM^–1^ cm^–2^ (Figure 13b), which we attribute mainly to matrix effects and competing ions rather than crosstalk between the analytes. To validate the method, results from real samples were compared with ICP–MS. Parallel ICP–MS analysis of the same known Pb^2+^ and Cd^2+^ concentrations in real matrices showed close agreement with the electrode-based measurements (91–94%). The calibration parameters obtained for Pb^2+^ determination in PBS (pH = 4) and real sample are summarized in Table 4. Both plots exhibit good linearity (*R* ^2^ > 0.98) within the 1–6 μM range. The lower slope observed for the real sample reflects a slight decrease in sensitivity, likely due to matrix effects that partially hinder Pb^2+^ diffusion or interaction with the electrode surface.

**Figure 13:**
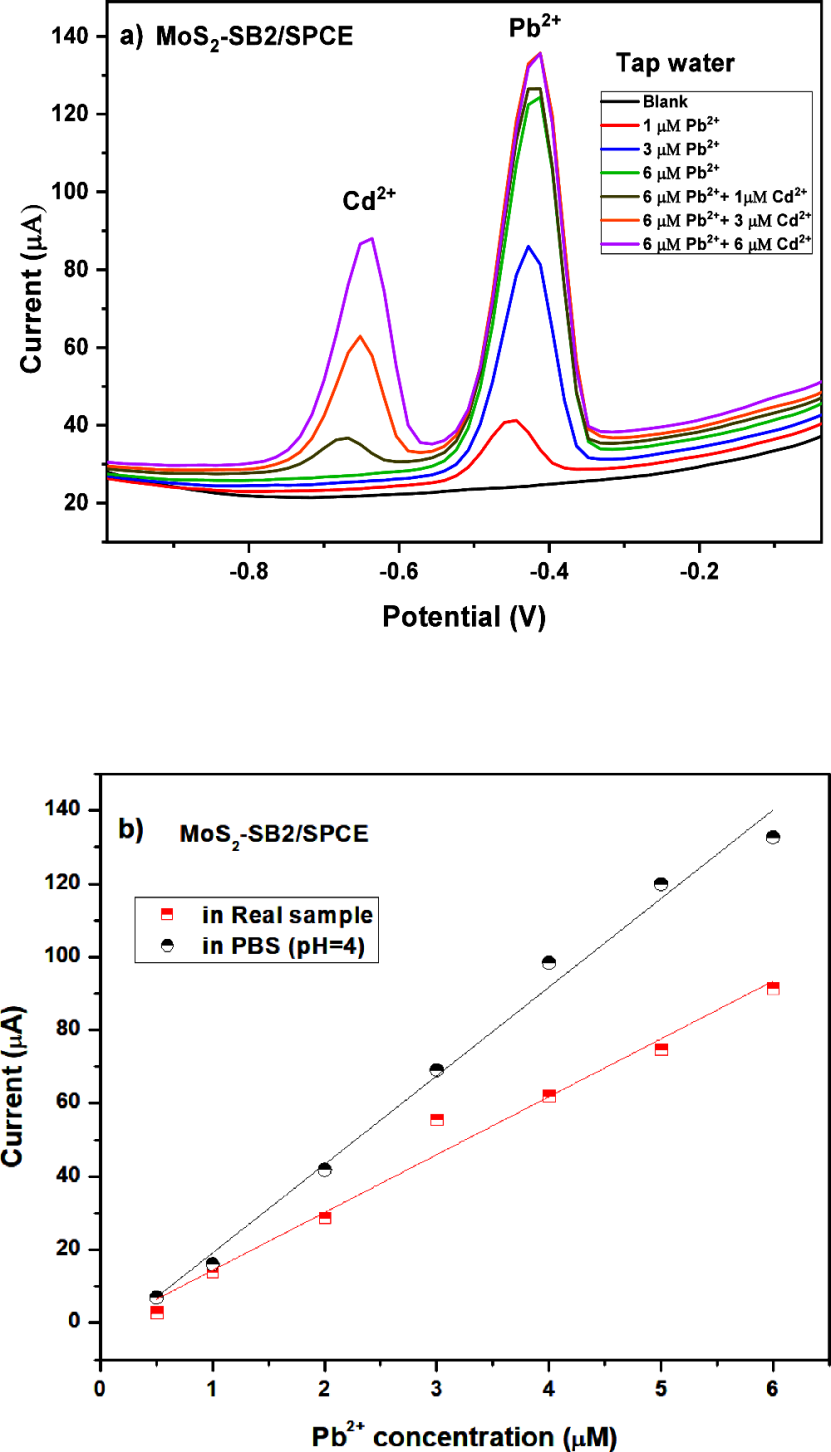
(a) Simultaneous determination of Cd^2+^ and Pb^2+^ on MoS_2_–SB2/SPCE using SWASV in tap water real sample and (b) calibration curve toward lead ions in PBS and real sample.

**Table 4:** Summary of the Calibration Parameters Obtained from the Calibration Plots of the MoS_2_–SB_2_/SPCE Sensor for Pb^2+^ Determination in PBS (pH = 4) and in the Real Sample

medium	linear range (μM)	calibration equation (*I* = *a* + *bC*)	*R* ^2^	sensitivity (μA μM^–1^)
PBS (pH = 4)	1–6	I = −5.043 + 24.203 *C*	0.991	24.20
real sample	1–6	I = −1.396 + 15.804 *C*	0.981	15.80

## 4 Conclusions

In this study, we developed a covalently conjugated MoS_2_–Schiff base interface (SB1 and SB2) to modify the working electrode of disposable screen-printed carbon electrodes (SPCEs). Both Schiff bases were successfully incorporated into MoS_2_-based nanocomposites, with MoS_2_@SB2 exhibiting the best electrochemical performance. Under optimized SWASV conditions in PBS at pH 4, the MoS_2_@SB2/SPCE achieved a sensitivity of 220.344 μA μM^–1^ cm^–2^ and a limit of detection of 0.267 μM within a 1–5 μM linear range for Pb^2+^. The strong electroanalytical response arises from the synergy between the high surface area and conductivity of MoS_2_ and the chelating/electron-donating properties of SB2, which together enhance charge transfer. Importantly, the platform provides simultaneous, well-resolved Pb^2+^/Cd^2+^ stripping peaks, while quantitative calibration is optimized for Pb^2+^. The sensor also shows good selectivity, high device-to-device reproducibility, and reliable performance in tap water samples. Alongside these advantages, the current implementation requires an acidic medium and a 180 s preconcentration step, and its primary linear range and LoD do not yet reach ultratrace benchmarks Nevertheless, the covalent interfacial design on a disposable transducer offers a simple and scalable route to heavy-metal monitoring. Future work will target extending the linear range and lowering the LoD, enhancing performance in complex matrices through electrolyte optimization and control of competitive binding at the interface, broadening interference studies, and assessing storage stability and on-chip integration toward portable field use.

## Supplementary Material



## Notes

^1^H NMR spectrum of 4-aminocinnamaldehyde, SB1 and SB2; ^13^C NMR of SB1 and SB2; EIS Nyquist plot (Exp vs Fit) for bare/SPCE, MoS_2_/SPCE, MoS_2_-MCA/SPCE, MoS_2_–SB1/SPCE and MoS_2_–SB2/SPCE; calibration curves of Pb^2+^ in the simultaneous determination with Cd^2+^ of MoS_2_–SB2/SPCE; and representative scanning electron microscopy/energy-dispersive X-ray (SEM/EDX) analyses of (a) Bare/SPCE and (b) MoS_2_/SPCE (PDF)
