# Supplementary material for: Enhanced Electrochemical Detection of Lead Ions Using Schiff Base/MoS2 Modified Screen-Printed Electrodes
Source: ACS Omega. 2025 Nov 11;10(48):58747–62. doi: 10.1021/acsomega.5c07147 (PMC12771212; doi:10.1021/acsomega.5c07147)
Supplement: Supplementary file 1 [file ao5c07147_si_001.pdf]

Supplementary Materials to:

Enhanced Electrochemical Detection of Lead ions using Schiff Base/MoS_2 modified Screen-Printed Electrodes

Daniela Iannazzo [a], Zahra Akbari [a], Consuelo Celesti [a*], Federica Bucolo [a*], Salvatore V. Giofrè [b], Sebastiano Vasi [c], Dario Morganti [d], Khouloud Abid [e], and Giovanni Neri [a]

[a] Department of Engineering, University of Messina, 98166, Messina, Italy

[b] Department CHIBIOFARAM, University of Messina, 98166, Messina, Italy

[c] Department MIFT, University of Messina, 98166, Messina, Italy

[d] IMM CNR, 95121, Catania, Italy

[e] CNR ITAE, viale F. Stagno D'Alcontres 37, Messina, I-98156, Italy

* Correspondence to: ccelesti@unime.it; fbucolo@unime.it

CONTENT:

Figure S1. ^{1}H NMR spectrum of 4-aminocinnamaldehyde……………………………pag. S2

Figure S2. ^{1}H NMR spectrum of **SB1**……………………………………………pag. S2

Figure S3. ^{13}C NMR spectrum of **SB1**……………………………………………pag. S3

Figure S4. ^{1}H NMR spectrum of **SB2**……………………………………………pag. S3

Figure S5. ^{13}C NMR spectrum of **SB2**……………………………………………pag. S4

Figure S6. EIS Nyquist plot (Exp vs. Fit) for bare/SPCE…………………………pag. S4

Figure S7. EIS Nyquist plot (Exp vs. Fit) for MoS_2/SPCE……………………….......pag. S5

Figure S8. EIS Nyquist plot (Exp vs. Fit) for MoS_2-MCA/SPCE…………………pag. S5

Figure S9. EIS Nyquist plot (Exp vs. Fit) for MoS_2-SB1/SPCE……………………...pag. S5

Figure S10. EIS Nyquist plot (Exp vs. Fit) for MoS_2-SB2/SPCE…………………pag. S6

Figure S11. Calibration curves in the simultaneous Pb^{2+}/Cd^{2+} detection …………………..pag. S6

Figure S12. SEM/EDX images of a) Bare/SPCE and b) MoS_2/SPCE………………………pag.S7

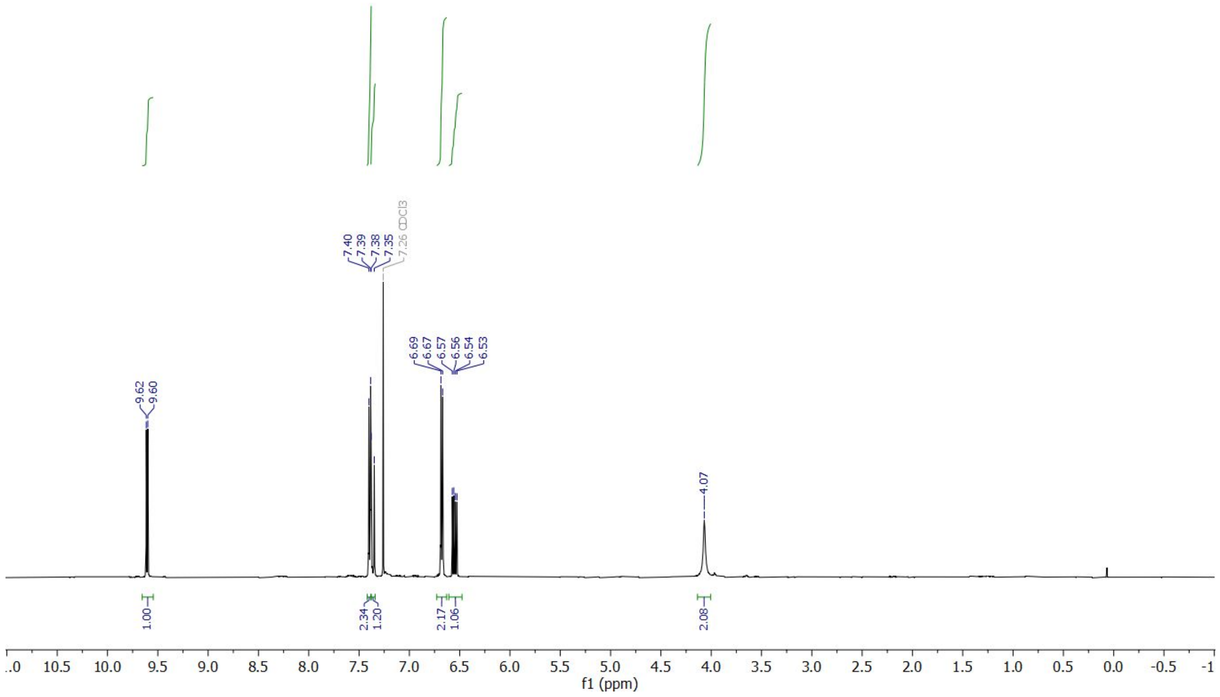

Figure S1. ^{1}H NMR spectrum of *4-aminocinnamaldehyde* in $CDCl_3$, recorded at 25°C and 500 MHz.

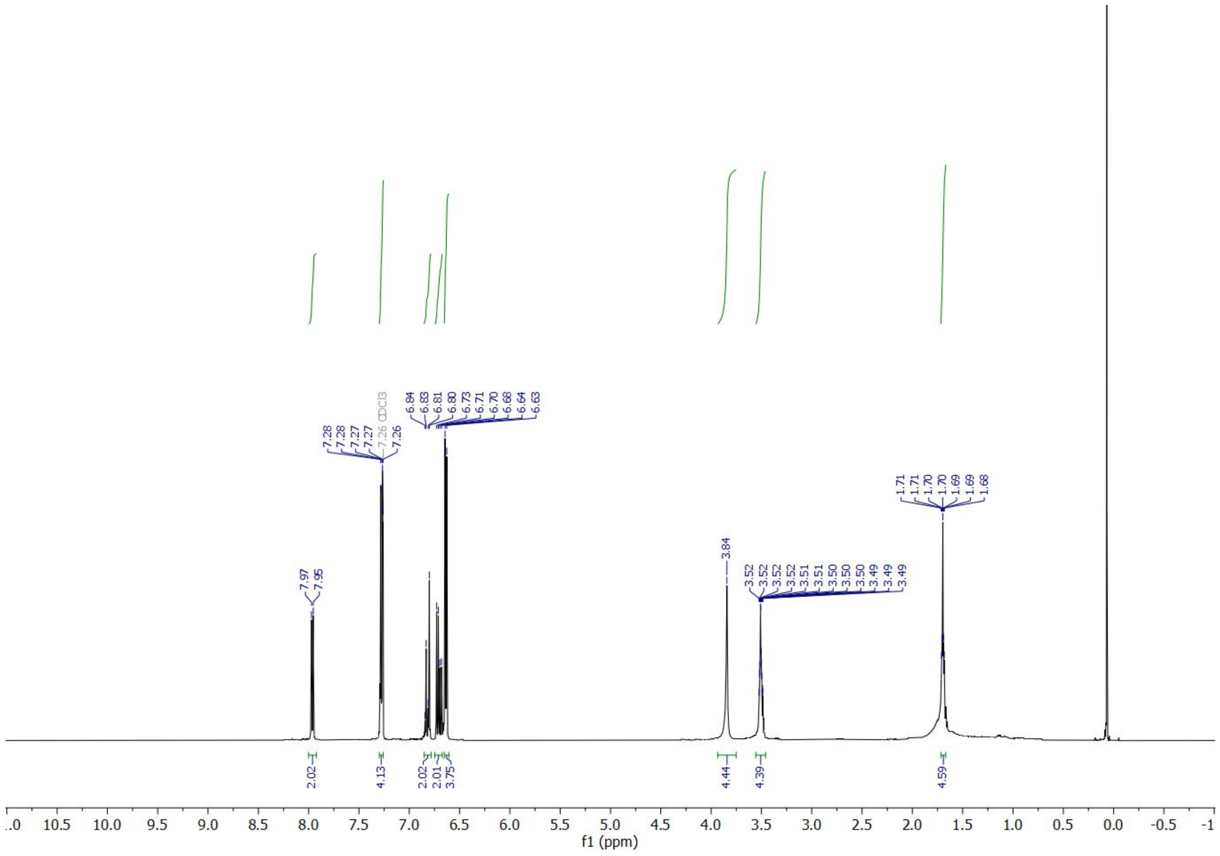

Figure S2. ^{1}H NMR spectrum of **SB1** in $CDCl_3$, recorded at 25°C and 500 MHz.

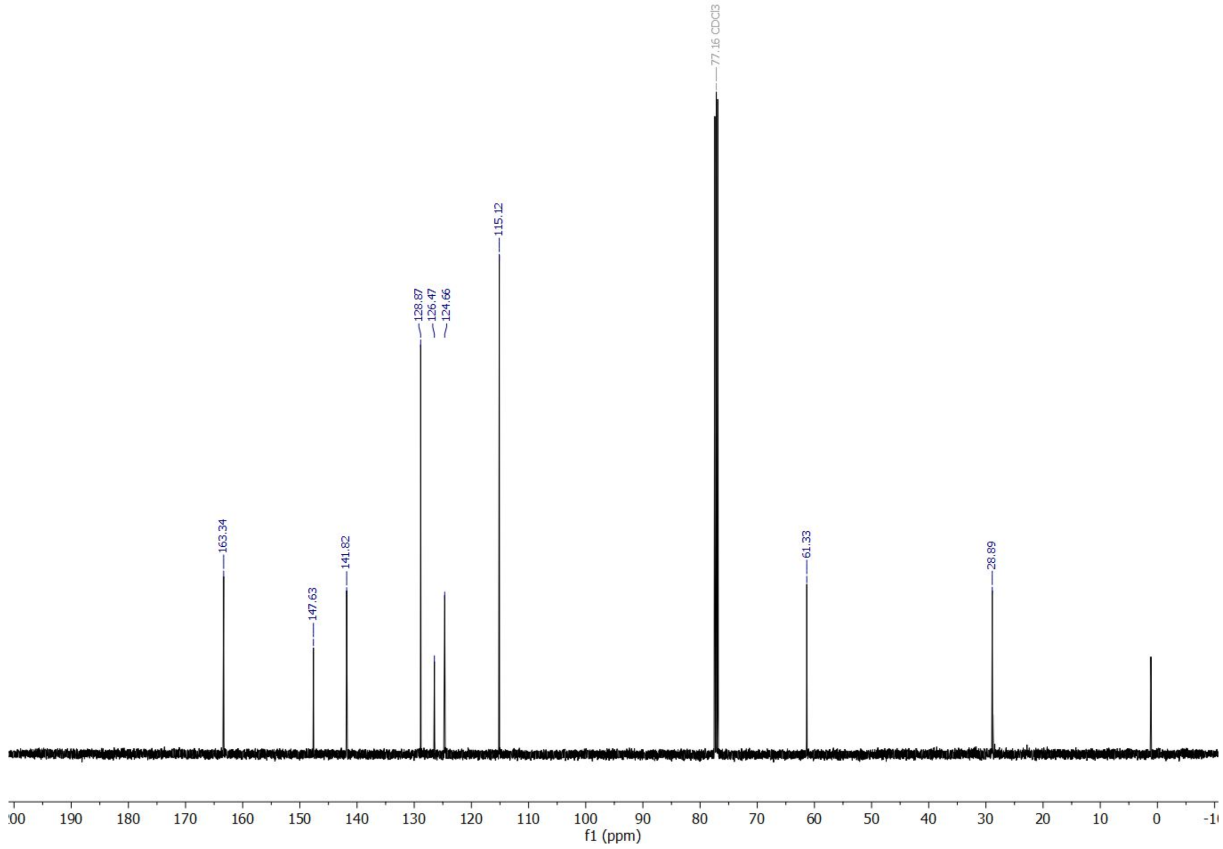

Figure S3. ^{13}C NMR spectrum of **SB1** in $CDCl_3$, recorded at 25°C and 126 MHz.

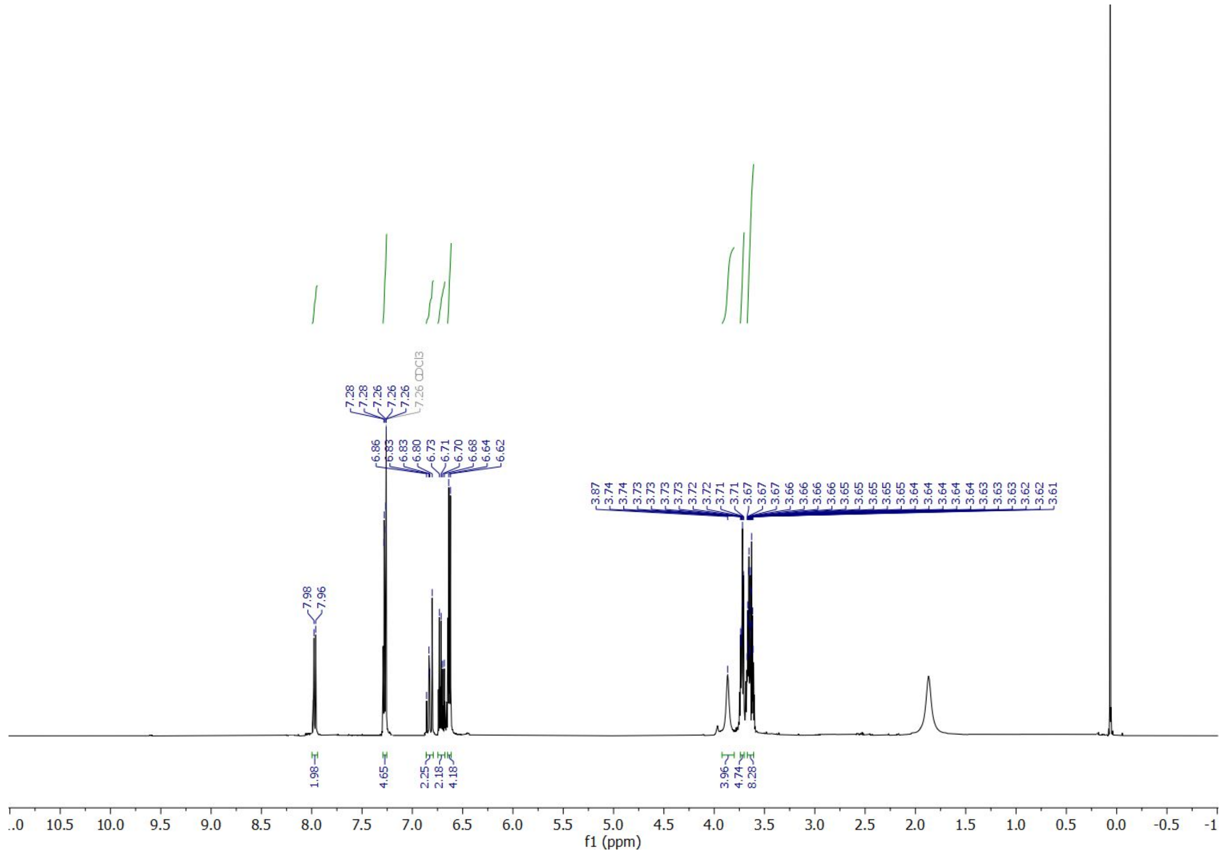

Figure S4. ^{1}H NMR spectrum of **SB2** in $CDCl_3$, recorded at 25°C and 500 MHz.

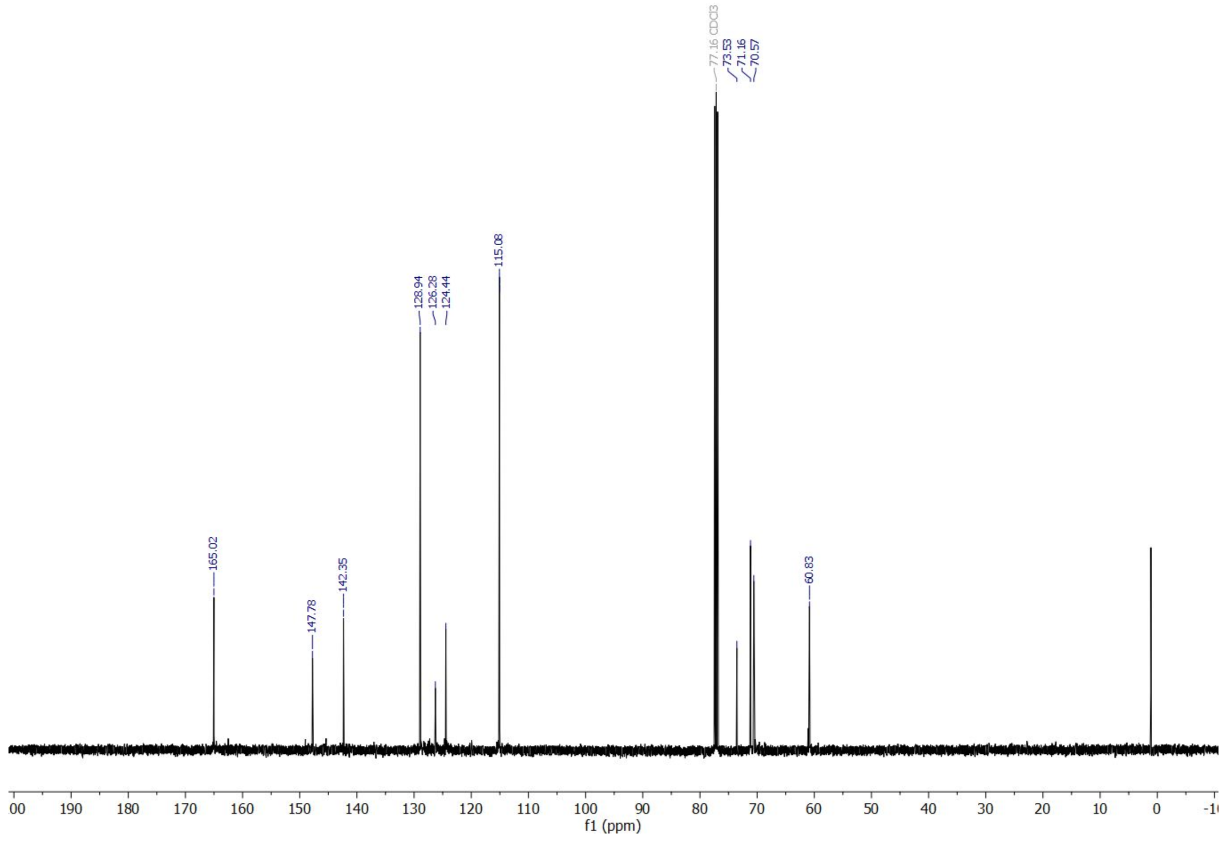

Figure S5. ^{13}C NMR spectrum of **SB2** in $CDCl_3$, recorded at 25°C and 126 MHz.

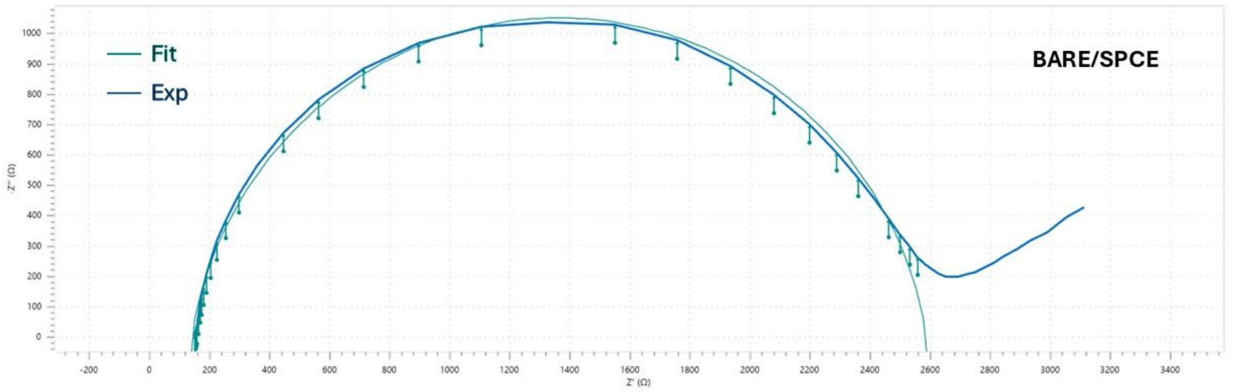

Figure S6. Nyquist plot showing experimental (Exp) and fitted (Fit) EIS data for the bare/SPCE

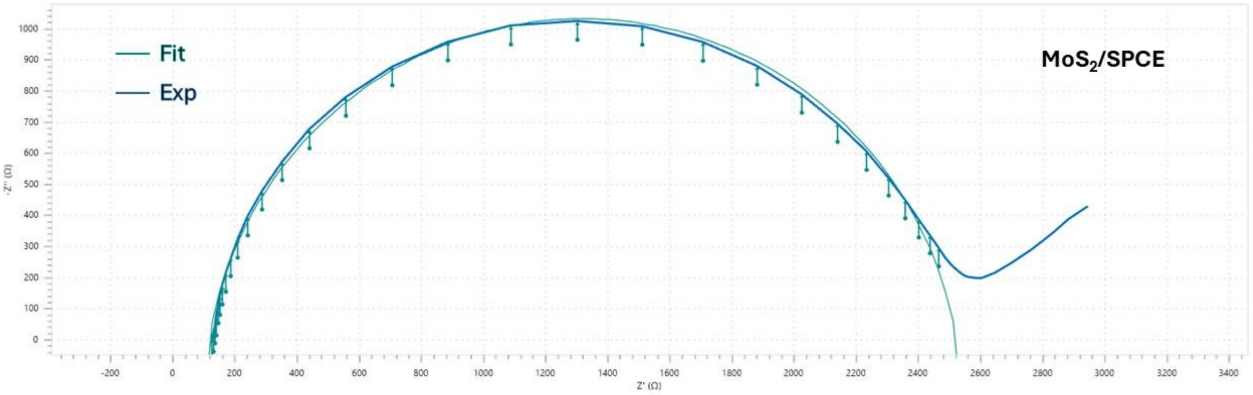

Figure S7. Nyquist plot showing experimental (Exp) and fitted (Fit) EIS data for the MoS_2/SPCE

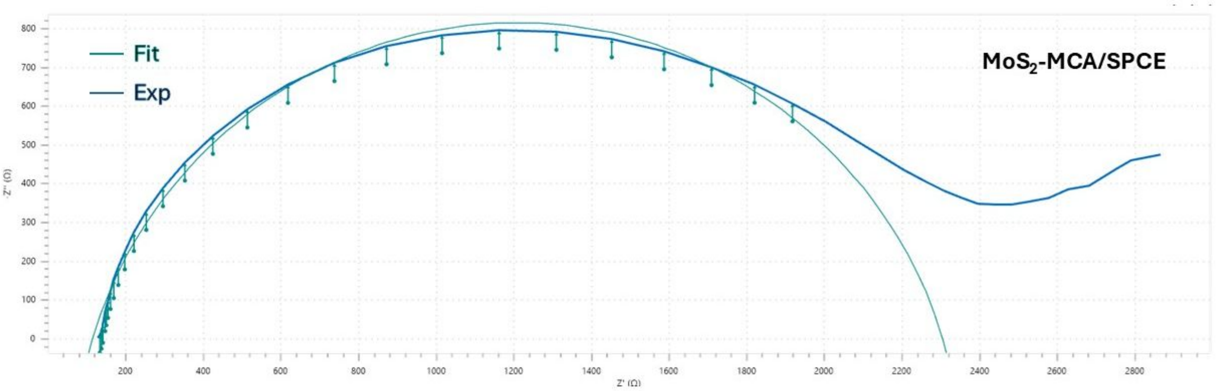

Figure S8. Nyquist plot showing experimental (Exp) and fitted (Fit) EIS data for the MoS_2-MCA/SPCE

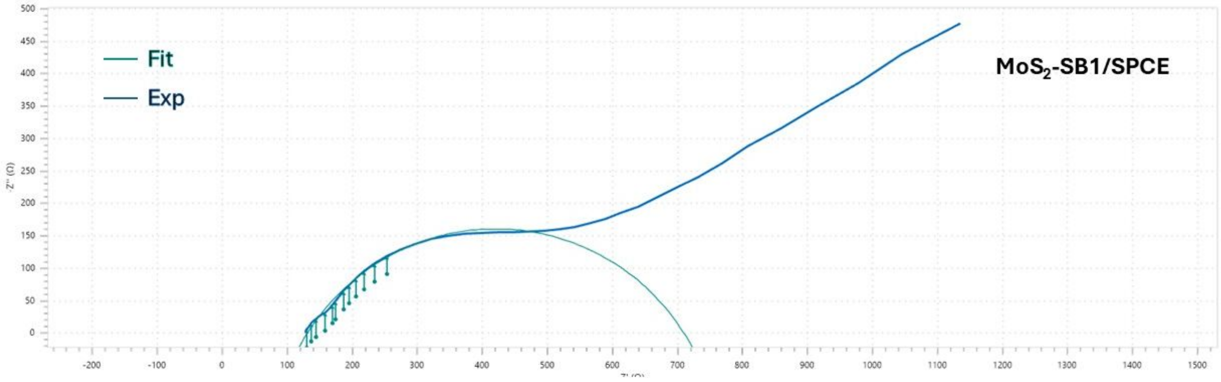

Figure S9. Nyquist plot showing experimental (Exp) and fitted (Fit) EIS data for the MoS_2-SB1/SPCE

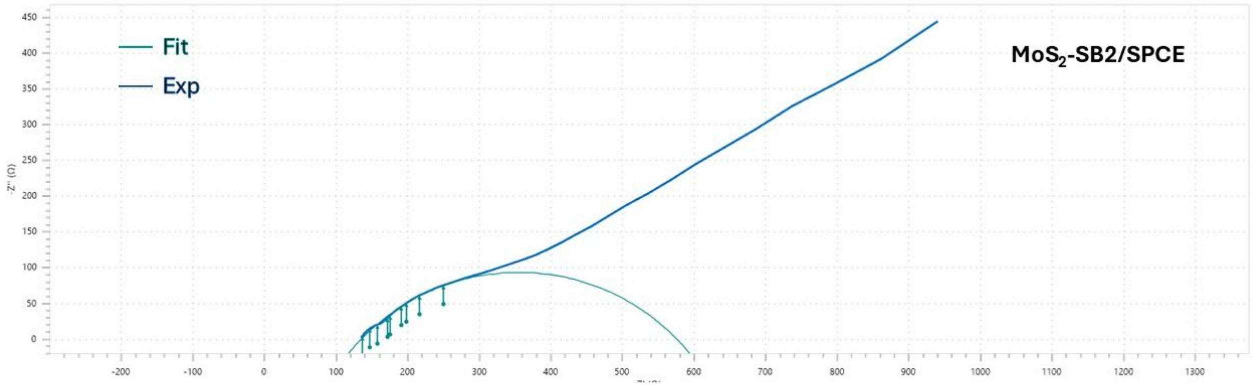

Figure S10. Nyquist plot showing experimental (Exp) and fitted (Fit) EIS data for the MoS_2-SB2/SPCE

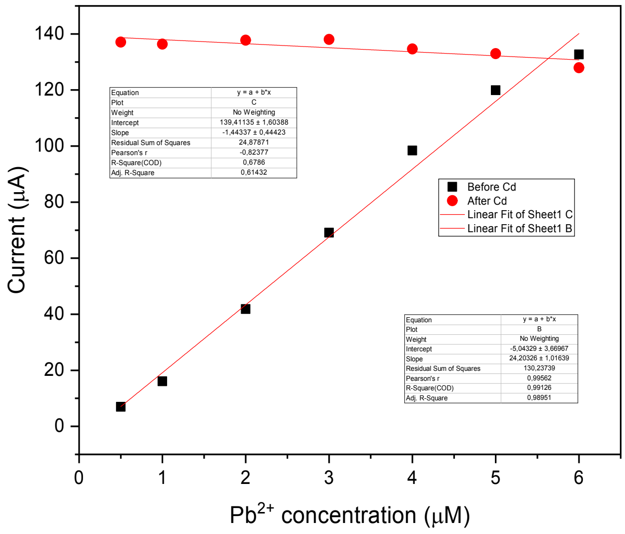

Figure S11. Calibration curves of Pb^{2+} in the simultaneous determination with Cd^{2+} of MoS_2-SB2/SPCE

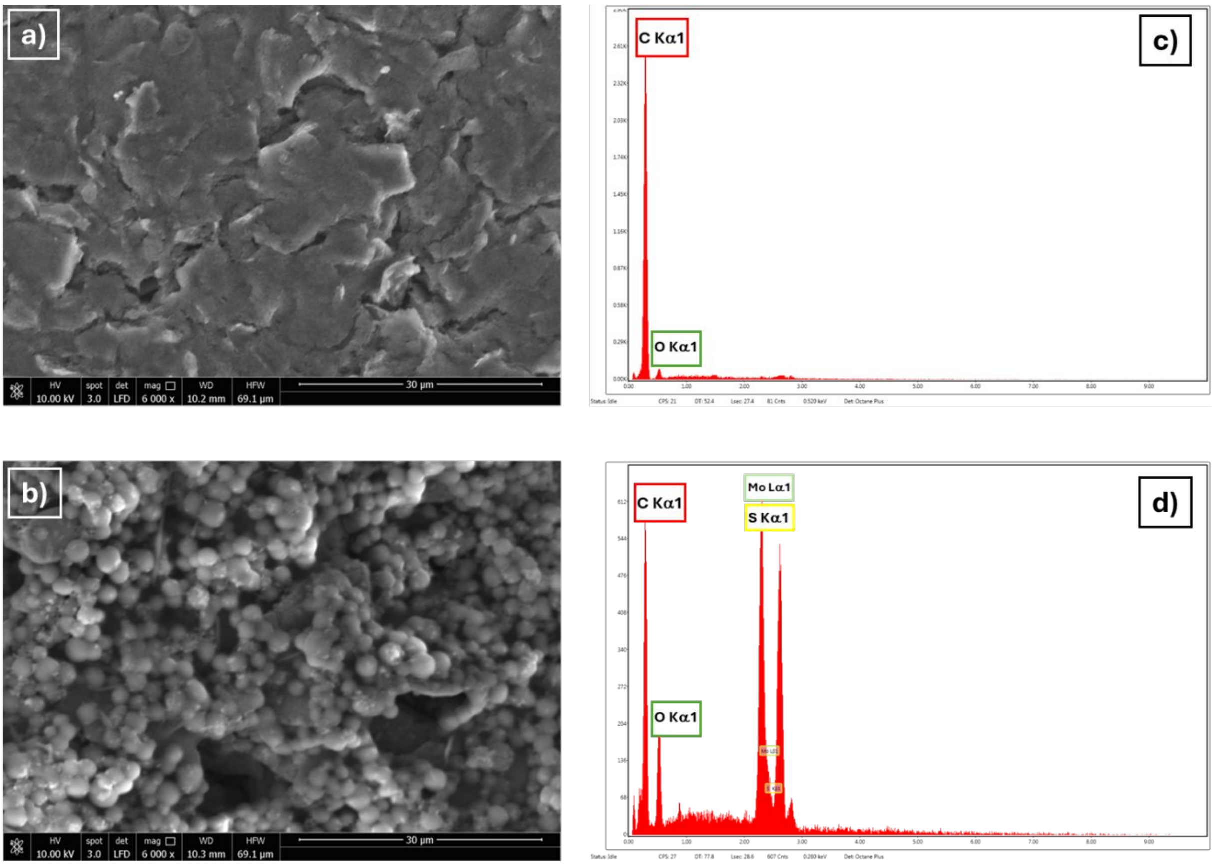

Figure S12. Representative scanning electron microscopy/energy-dispersive X-ray (SEM/EDX) analyses of a) Bare/SPCE and b) MoS_2/SPCE
